# Detection of Independent Associations of Plasma Lipidomic Parameters with Insulin Sensitivity Indices Using Data Mining Methodology

**DOI:** 10.1371/journal.pone.0164173

**Published:** 2016-10-13

**Authors:** Steffi Kopprasch, Srirangan Dheban, Kai Schuhmann, Aimin Xu, Klaus-Martin Schulte, Charmaine J. Simeonovic, Peter E. H. Schwarz, Stefan R. Bornstein, Andrej Shevchenko, Juergen Graessler

**Affiliations:** 1 Department of Internal Medicine 3, University Hospital Carl Gustav Carus, Technische Universität Dresden, Dresden, Germany; 2 Institute for Medical Informatics and Biometry, Faculty of Medicine Carl Gustav Carus, Technische Universität Dresden, Dresden, Germany; 3 Max Planck Institute of Molecular Cell Biology and Genetics, Dresden, Germany; 4 Department of Medicine, Li Ka Shing Faculty of Medicine, The University of Hong Kong, Hong Kong, China; 5 Department of Endocrine Surgery, King’s College Hospital, NHS Foundation Trust, London, United Kingdom; 6 Department of Immunology and Infectious Disease, The John Curtin School of Medical Research, The Australian National University, Canberra, Australia; Virgen Macarena University Hospital, School of Medicine, University of Seville, SPAIN

## Abstract

**Objective:**

Glucolipotoxicity is a major pathophysiological mechanism in the development of insulin resistance and type 2 diabetes mellitus (T2D). We aimed to detect subtle changes in the circulating lipid profile by shotgun lipidomics analyses and to associate them with four different insulin sensitivity indices.

**Methods:**

The cross-sectional study comprised 90 men with a broad range of insulin sensitivity including normal glucose tolerance (NGT, n = 33), impaired glucose tolerance (IGT, n = 32) and newly detected T2D (n = 25). Prior to oral glucose challenge plasma was obtained and quantitatively analyzed for 198 lipid molecular species from 13 different lipid classes including triacylglycerls (TAGs), phosphatidylcholine plasmalogen/ether (PC O-s), sphingomyelins (SMs), and lysophosphatidylcholines (LPCs). To identify a lipidomic signature of individual insulin sensitivity we applied three data mining approaches, namely least absolute shrinkage and selection operator (LASSO), Support Vector Regression (SVR) and Random Forests (RF) for the following insulin sensitivity indices: homeostasis model of insulin resistance (HOMA-IR), glucose insulin sensitivity index (GSI), insulin sensitivity index (ISI), and disposition index (DI). The LASSO procedure offers a high prediction accuracy and and an easier interpretability than SVR and RF.

**Results:**

After LASSO selection, the plasma lipidome explained 3% (DI) to maximal 53% (HOMA-IR) variability of the sensitivity indexes. Among the lipid species with the highest positive LASSO regression coefficient were TAG 54:2 (HOMA-IR), PC O- 32:0 (GSI), and SM 40:3:1 (ISI). The highest negative regression coefficient was obtained for LPC 22:5 (HOMA-IR), TAG 51:1 (GSI), and TAG 58:6 (ISI).

**Conclusion:**

Although a substantial part of lipid molecular species showed a significant correlation with insulin sensitivity indices we were able to identify a limited number of lipid metabolites of particular importance based on the LASSO approach. These few selected lipids with the closest connection to sensitivity indices may help to further improve disease risk prediction and disease and therapy monitoring.

## Introduction

Metabolic syndrome represents a cluster of metabolic and cardiovascular abnormalities including obesity, hypertension, hyperglycemia and dyslipidemia. A common pathophysiological mechanism linking these traits is insulin resistance which in turn is closely associated with abnormalities in glucose and lipid metabolism.

Intracellular lipid oversupply may lead to cellular damage that underlies diabetes [1–3]. So-called glucolipotoxicity with accompanying insulin resistance is followed not only by disturbances in tissue fat metabolism but is also reflected in alterations of various circulating plasma/serum lipid subspecies [4–7]. Recently, we demonstrated that hypertension, an integral part of the metabolic syndrome, was specifically associated with decreased levels of free cholesterol and ether lipids [8].

The objective of this study was to apply shotgun mass spectrometric analysis [9, 10] for the comprehensive and quantitative estimation of the plasma lipidomic profile in a total of 90 male individuals with a broad range of insulin sensitivity including NGT (n = 33), IGT (n = 32) and newly detected T2D (n = 25). The collection of high dimensional data which contains a large number of potential covariates, yet a very limited sample size is often called “small n and large p problem”. This requires specific statistical data mining methods for detecting independent associations between molecular lipid species and insulin sensitivity indices including HOMA-IR, GSI, ISI, and DI. Here, we demonstrate that LASSO selection procedure was a valuable tool to detect distinct lipidomic signatures of the four investigated insulin sensitivity indexes. Their close association with different insulin sensitivity indices may link these molecular markers with subtle changes of glucose tolerance.

## Research Design and Methods

### Subjects

The study comprised 90 white male subjects who were attending the PRAEDIAS (Prevention of diabetes) study at the Dresden University Hospital Carl Gustav Carus. In brief, subjects who were at risk for development of diabetes owing to a family history of T2D, obesity and/or hyper-/dyslipoproteinemia were examined. Clinically overt diabetes was an exclusion criterion. The study was conducted in accordance with the guidelines proposed in the Declaration of Helsinki. All subjects signed a written consent to participate in the study. The study was approved by the local competent authority the Ethik-Kommission an der Technischen Universität Dresden (EK 139092001).

After overnight fasting subjects underwent a comprehensive clinical and metabolic characterization including an oral glucose tolerance test (OGTT, 75g oral glucose challenge). Accordingly, study participants were grouped: plasma glucose at time point 0 (PG0) <6.1 mmol/l and 2h post challenge (PG120) <7.8 mmol/l—NGT; PG120 = 7.8–11.1 mmol/l—IGT; PG120>11.1 mmol/l—T2D. Subjects included into the latter group were diagnosed by their first pathological OGTT. Notably, they had no clinical symptoms and no medication of diabetes mellitus.

Blood samples for lipid profiling were taken at time point 0 of OGTT. EDTA plasma was prepared by centrifugation at 4°C and 3000g for 10 min, immediately shock-frozen in liquid nitrogen and stored at -80°C until analysis.

### Conventional clinical chemistry

Plasma total cholesterol, HDL and LDL cholesterol, triglycerides, free fatty acids, glucose, insulin, C-peptide, leukocytes, C-reactive protein (CRP), and HBA_1c_ were measured by routine clinical chemistry as previously described [8].

### Calculation of insulin sensitivity indices

The sensitivity indices HOMA-IR, ISI, and DI were estimated as described previously [11]. GSI was introduced recently by Kazama et al. [12] and is calculated from plasma glucose and insulin levels at 0, 30, 60, 90 and 120 min OGTT by a formula based on an autoregressive model, see also S1 Table.

### Shotgun lipidomics analysis

#### Chemicals and lipid standards

Common chemicals and solvents were of ACS or LC–MS grade (purity > 99.0%) and purchased from Fisher Scientific (Loughborough, United Kingdom), Sigma–Aldrich Chemie GmbH (Munich, Germany), Fluka (Buchs St. Gallen, Switzerland) or Merck (Darmstadt, Germany). Synthetic standards from Avanti Polar Lipid Inc. (Alabaster, AL) were used for quantification (S2 Table).

#### Lipid extraction

EDTA blood plasma samples were extracted similar to previously published methods [8, 13]. Briefly, in a 1.5 ml “Eppendorf Safe-Lock” tube (Eppendorf (Hamburg, Germany)) 5 μl of plasma were mixed with 700 μl of MTBE/methanol 10:3 (v/v) containing internal standards (S2 Table). The mixture was vortexed for one hour at 4°C. 140 μl water were added and the tube vortexed for another 15 min at 4°C. After centrifugation for 5 min at 13.400 rpm on a Minispin centrifuge (Eppendorf, Hamburg, Germany), 500 μl the upper organic phase were transferred into 1.5 ml borosilicate vials and stored at −20°C until analysis.

#### Mass spectrometric analysis

Shotgun Lipidomics measurements were performed similar to previous methods (doi: 10.1002/jms.2031). Briefly, 5 μl of plasma extract were diluted in 100 μl chloroform/methanol/2-propanol 1/2/4 (v/v/v) containing 7.5 mM ammonium formate. Measurements were performed on a QExactive mass spectrometer (Thermo Fisher Scientific, Bremen, Germany) equipped with a robotic nanoflow ion source TriVersa (Advion BioSciences Ltd, Ithaca NY) (similar to [9]). Dual polarity acquisitions were performed with ionization voltage ± 0.95 kV and the gas pressure to 1.25 psi. FT MS spectra (*m/z* 400–1200, AGC = 1e6, IT = 500ms) were acquired at the targeted resolution of 140k at *m/z* 200 in both polarity modes. Each lipid extract was twice analyzed. Cholesterol was quantified by targeted FT MS/MS+ (AGC = 5e4, IT = 3s) at the targeted resolution of 140k at *m/z* 200 (as described elsewhere).

#### Performance control

A series of control blood plasma (sample volume: 1.25, 2.5, 5.0, 10.0, 15.0 μl) was measured to prove detection linearity of quantified lipid species for the expected total lipid concentrations of 2.0–28.3 mM. For the analyzed samples of this screen the total lipid concentration in blood plasma varied from 5.6–18.7 mM. Within the tested concentration range the adjusted R^2^ was greater 0.98 for the quantified lipid analytes.

Total triacylglycerol and cholesterol concentrations, as specified by lipidomics, were correlated with quantities obtained by standard enzymatic methods on a MODULAR analyser (Roche, Indianapolis, IN). The specified concentrations correlated well for total triacylglycerol (relative error = -5.48 ± 8.77%, adj. R^2^ = 0.9754, slope = 1.0151) and total cholesterol (relative error = 7.61 ± 5.44%, adj. R^2^ = 0.9362, slope = 0.9833). The data comparison uncovered 1 experimental outlayer in 91 analyzed plasma samples. For this sample total triglyceride differed by 7.9x; total cholesterol by 1.6x.

#### Data processing

FT MS and FT MSMS spectra were interpreted by LipidXplorer software as described [9, 14]. 198 species from 13 major lipid classes were quantified across the plasma samples (S2 Table).

#### Annotation of lipid species

Lipid species were annotated according to common rules (DOI 10.1194/jlr.M033506). Abbreviation of tri- and diacylglycerols were different to avoid confusion with colorimetric specified quantitities for same lipid classes. Further, sphingolipid hydroxyl groups are assigned by separate counter following the double bond counter. Brief description of short annotation for lipid species: <lipid class abbreviation> <number of chain carbon atoms>: <number of chain double bonds>: <number of hydroxyl groups (if present)>.

#### Desaturase activities

The ratios of product-to precursor fatty acids in plasma cholesterol esters were used to estimate desaturase activities: Δ5-desaturase (D5D): arachidonic acid (FA 20:4 *n*-6)/dihomo-γ-linolenic acid (FA 20:3 *n*-6); Δ6-desaturase (D6D): γ-linolenic acid (FA 18:3 *n*-6)/linoleic acid (FA 18:2 *n*-6); Δ9-desaturase (D9D): palmitoleic acid (FA 16:1 *n*-7)/palmitic acid (FA 16:0).

#### Statistical analysis

The initial statistical analysis focused on detecting group effects on the lipid species as well as the lipid classes. As the group variable contains the NGT, IGT and T2D groups an ANOVA was used to identify differences in group means after having log-transformed and normalized the lipid values. In total, 202 p-values were returned that were corrected for multiple testing using the Benjamini-Hochberg procedure which is a preferred correction procedure analyzing high dimensional data. As an ANOVA is only able to determine whether one of the group means significantly differs from the others, these calculations were followed by pairwise t-Tests in order to conclude which of the group means differ significantly. Here we also used a Benjamini Hochberg correction to account for multiple testing. Similarly, correlations of each insulin sensitivity index and lipid species was estimated using the Spearman coefficient followed by correlation tests. As the tests were performed for each of the lipids in combination with each of the insulin sensitivity indices this also produces a multiple testing problem which was again addressed by a Benjamini-Hochberg correction.

In order to estimate the influence of lipids on four insulin sensitivity indices (HOMA-IR, GSI, DI and ISI) in a multivariate sense we only included either all lipid species or all lipid classes as covariates in our models. The analysis was corrected for age, BMI, WHR and systolic blood pressure including these covariates in the models explaining each of the four insulin sensitivity indices.

The huge number of covariates, however, makes it impractical to use standard parametric procedures such as linear regression from a technical point of view having only observed 90 individuals, on the other hand the interpretation of such a model would be almost impractical due to a huge number of estimated regression coefficients. In practice many features may be irrelevant and only a few covariates will actually influence the response. Including irrelevant features to a model would then lead to overfitting as these variables can be considered as noise variables that will cover the signal which would cause better fits on training samples and poorer performances on test samples. Hence, including irrelevant features would lead to a model that would not be able to generalize the trend in the data due to the noise variables.

Thus, data mining tools were applied to the data at hand that permit a fair analysis of such data. We used LASSO [15], Support Vector Regression (SVR, [16]) in combination with a p-value based feature selection using correlation tests as well as a Benjamini-Hochberg correction prior to applying the SVR and Random Forests for each of the four regression tasks. In order to get an overview on Random Forests as well as variable importance which are major outcomes of this method we refer to Strobl et al. [17]. These three methods were validated using a cross-validation technique called Monte Carlo Cross Validation with 100 iterations [18]. After validation we selected the model that minimizes the mean absolute error on the test data as the model with the lowest error is the most valid. However, it turned out that the LASSO model either returned the lowest mean absolute error or performed as good as the other methods. In the latter case we decided to proceed with the LASSO model as it was as valid as the other two models, however easier to interpret.

Finally, we extracted the final models for each of the four sensitivity indices by running the method that is the most valid according to the mean absolute error on the entire data set and estimating the final regression coefficients as well as R-Squared statistics for all four regression models.

## Results

Baseline anthropometric data and glycemic status are presented in Table 1. Compared to NGT individuals, patients with newly diagnosed T2D were older, had a significantly increased WHR and a higher systolic blood pressure. According to WHO criteria (RR≥140/90) fifty one individuals were hypertensive (thirteen with NGT, nineteen with IGT, nineteen with T2D). IGT and diabetic subjects had higher HbA_1c_, plasma glucose, insulin and C-peptide levels. Insulin sensitivity indices GSI, ISI and DI decreased, and HOMA-IR increased with increasing insulin resistance among individuals (see Table 1). While all sensitivity indices were significantly different between NGT and T2D subjects, ISI and DI additionally differed significantly between NGT and IGT individuals. As expected by us, the indices are closely related among themselves. Correlation analysis revealed that, except DI and HOMA-IR as well as DI and GSI, the indices were significantly associated (Spearman-rho correlation coefficients from -0.676 to 0.575).

**Table 1:** Baseline clinical data, glycemic status, and conventional lipid parameters of NGT, IGT, and diabetic (T2D) male subjects.

Parameter	NGT	IGT	T2D	p-values		
	(n = 33)	(n = 32)	(n = 25)	*IGT/NGT*	*T2D/NGT*	*IGT/T2D*
**Clinical data**						
Age (years)	54±2	60±2	63±2	0.192	0.017	0.861
BMI (kg/m²)	28.3±0.8	29.0±0.7	30.4±0.7	1.000	0.165	0.597
WHR	0.94±0.01	0.97±0.01	1.00±0.01	0.243	0.001	0.122
Blood pressure (mmHg)						
systolic	131±3	143±3	151±4	0.016	0.000	0.430
diastolic	78±1	85±2	85±2	0.038	0.074	1.000
CRP (mg/l)	1.3±0.2	1.6±0.2	2.0±0.3	1.000	0.100	0.649
Leukocytes (GPt/l)	5.5±0.3	5.8±0.3	5.8±0.2	1.000	1.000	1.000
**Glycemic status**						
HbA1C (mmol/mol)	36±1	40±1	44±1	0.011	0.000	0.010
Plasma glucose (mmol/l)						
0 min	5.24±0.08	5.73±0.10	6.81±0.18	0.009	0.000	0.000
120 min	5.06±0.21	8.95±0.14	12.06±0.49	0.000	0.000	0.000
Insulin (pmol/l)						
0 min	79.9±18.0	79.7±10.3	142.4±18.3	1.000	0.000	0.024
120 min	213.6±35.5	647.3±85.4	774.2±105.4	0.000	0.000	0.078
C-peptide (pmol/l)						
0 min	841±71	870±43	1377±117	0.689	0.000	0.000
120 min	2200±166	3674±237	3979±279	0.000	0.000	1.000
**Insulin sensitivity indices**						
GSI^a^	0.89±0.15	0.63±0.11	0.29±0.05	0.379	0.004	0.186
ISI^b^	6.93±0.76	3.08±0.14	2.19±0.11	0.000	0.000	0.655
DI^c^	9710±1648	2804±321	2151±267	0.000	0.000	1.000
HOMA-IR^d^	2.81±0.68	2.97±0.38	3.81±0.39	1.000	0.001	0.002
**Conventional lipid parameters**						
Total cholesterol (mmol/l)	4.39±0.11	5.22±0.17	5.54±0.21	0.001	0.000	0.597
Triglycerides (mmol/l)	1.06±0.07	1.92±0.24	2.28±0.29	0.010	0.000	0.731
Free fatty acids (mmol/l)	0.40±0.03	0.49±0.03	0.61±0.17	0.035	0.000	0.018
HDL cholesterol (mmol/l)	1.40±0.05	1.28±0.06	1.19±0.09	0.340	0.034	0.849
LDL cholesterol (mmol/l)	2.58±0.09	3.19±0.14	3.48±0.20	0.005	0.000	0.447

Data are given as means ± SEM. Statistics by univariate analysis of variance with post-hoc Bonferroni test.

^a^–glucose insulin sensitivity index

^b^–insulin sensitivity index

^c^–disposition index

^d^–homeostasis model of insulin resistance

0 min and 120 min represents values before and after oral glucose challenge

Shotgun lipidomics allowed detection and absolute quantification of a total of 198 circulating lipid species covering 13 different lipid classes: CE (n = 13 species), PC (n = 27), TAG (n = 56), LPC (n = 13), LPE (n = 6), SM (n = 25), PI (n = 11), DAG (n = 6), PC O- (n = 15), PE (n = 10), PE O- (n = 11), Cer (n = 4), Chol (n = 1). Table 2 displays plasma concentration of lipid classes in plasma of individuals in the NGT, IGT and T2D groups, where the content of an individual lipid class was determined by summing up absolute concentrations of all identified species and is expressed as μmol/l. As shown in Table 2, cholesteryl ester were the most abundant among blood plasma lipids, followed by phosphatidylcholines, triacylglycerols, and free cholesterol consistent with a previous report [19].

**Table 2:** Circulating lipid status of NGT, IGT, and diabetic (T2D) male subjects.

Parameter	NGT	IGT	T2D	p-values		
	(n = 33)	(n = 32)	(n = 25)	*IGT/NGT*	*T2D/NGT*	*IGT/T2D*
**Mass spectrometric analysis (Sum lipid classes, μmol/l)**						
Triacylglycerols (TAG)	1065±87	1782±218	2237±323	0.045	0.001	0.440
Diacylglycerols (DAG)	39.6±	63.2±7.1	73.9±9.0	0.026	0.001	0.788
Cholesterylesters (CE)	3771±100	4330±122	4584±173	0.006	0.000	0.542
Ceramides (Cer)	4.95±0.26	6.22±0.37	7.31±0.47	0.032	0.000	0.132
Phosphatidylcholines (PC)	1074±26	1291±49	1377±45	0.000	0.000	0.455
Lysophosphatidylcholines (LPC)	273±7	302±15	290±11	0.203	0.944	1.000
Lysophosphatidyl-ethanolamines (LPE)	9.2±0.4	10.4±0.7	10.0±0.6	0.350	1.000	1.000
Ether-linked phosphatidyl-cholines (PC O-)	48.7±1.9	52.7±1.6	51.3±1.5	0.264	0.879	1.000
Phosphatidylinositols (PI)	37.2±1.4	49.0±2.8	53.6±2.9	0.001	0.000	0.573
Phosphatidylethanolamines (PE)	14.0±0.8	23.7±2.4	27.1±2.4	0.001	0.000	0.694
Ether-linked Phosphatidyl-ethanolamines (PE O-)	25.4±1.6	28.3±1.0	28.6±1.2	0.351	0.317	1.000
Sphingomyelins (SM)	352±8	372±9	389±15	0.489	0.050	0.802
Free cholesterol (Chol)	1047±32	1239±50	1350±62	0.011	0.000	0.340

Data are given as means ± SEM. P-values were adjusted by means of Benjamini-Hochberg procedure.

Out of 13 lipid classes, eight (TAG, DAG, CE, Cer, PC, PI, PE, Chol) were significantly and gradually increased in IGT and T2D subjects when compared with NGT individuals (Table 2). Additionally, SMs were significantly increased in T2D individuals when compared to NGT subjects. S3 Table shows the plasma concentrations of each lipid species in the three groups with distinct glucose tolerance and the p-values of differences between NGT, IGT, and T2D individuals.

**Fig 1:**
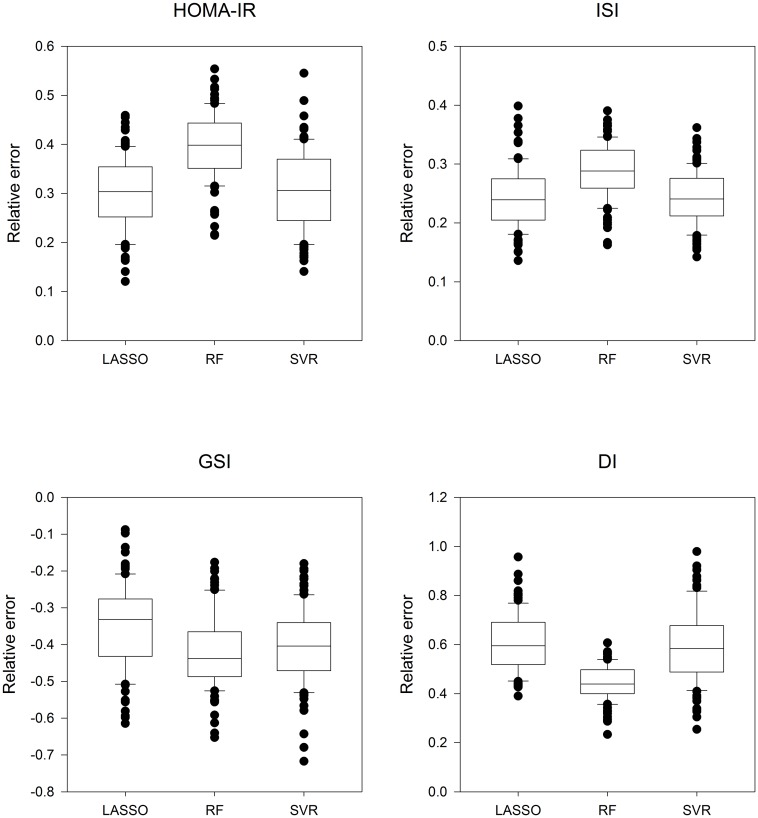
Relative errors of insulin sensitivity indices HOMA-IR, ISI, GSI, and DI explained by lipid species and age, BMI, WHR and systolic blood pressure. Three different selection methods were applied: LASSO, Random Forests (RF), and support vector regression (SVR). Data are presented as boxplots plus median.

Fig 1 presents the relative errors of insulin sensitivity indices using three regression models (LASSO, RF, and SVR) that included the common risk factors age, BMI, WHR, systolic blood pressure, and 198 individual lipid species. As shown in Fig 1, differences between these three approaches were only marginal. Due to better interpretability, however, in the present study the LASSO model was applied for subsequent analyses.

To unravel potential associations between circulating lipids and insulin sensitivity markers, we performed correlation analyses between lipids and four insulin sensitivity indices across all individuals using Spearman’s nonparametric rank correlation coefficient followed by correlation tests that have been adjusted for multiple testing using Benjamini Hochberg correction. As demonstrated S4 Table, out of 198 lipid molecular species 126 correlated significantly with either HOMA-IR, a marker of insulin resistance, and/or the surrogate sensitivity indexes GSI, ISI and DI. The most striking (as evaluated by rho, p-value, and number of lipid species within one lipid class) associations were found between HOMA-IR and TAGs (35 significant positive correlations), and ISI and TAGs (33 significant negative correlations). GSI and DI were significantly negatively associated with 21 TAGs and 14 TAGs, respectively. Therefore, it is not surprising that a considerable amount of lipid classes was either significantly positively (HOMA-IR) or significantly negatively (ISI, DI) related to insulin sensitivity indices, see S1 Fig. In this respect, the the causes for the comparably week performance of GSI remain to be determined.

S2 Fig shows a heat map of the logarithmic lipid class values in the rows and patients in the columns labelled according to their status of glucose tolerance (green = NGT, yellow = IGT and red = T2D). Most of the T2D and NGT subjects could be well separated by the lipid class values. They are either clustered on the left side of the heat map (T2D) or in the middle (NGT). However, it was not possible to detect a cluster of IGT patients indicating IGT patients can not be well separated from NGT or T2D subjects based on lipid class values.

**Fig 2:**
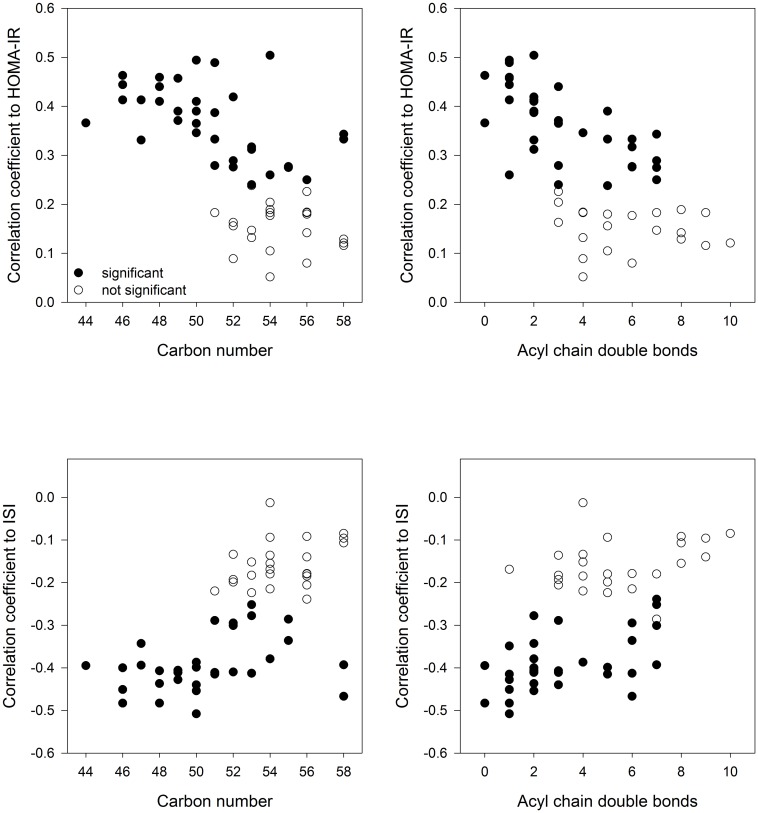
Spearman rank correlation of TAG species with different total acyl chain carbon numbers and double bond content and the insulin sensitivity indices HOMA-IR and ISI.

Fig 2 depicts the relationship of acyl chain carbons and acyl chain double bonds in TAGs and correlation coefficients to HOMA-IR and ISI across all individuals. It shows the tendency that TAGs of lower carbon number and double bond content were associated with increased positive correlation to insulin resistance (HOMA-IR) and increased negative association to insulin sensitivity (ISI). A similar relationship of TAG carbon number and acyl chain number with insulin sensitivity surrogates was also found for GSI but not for DI. Moreover, correlation analyses revealed a close positive association between ISI and ether lipids, LPCs and SMs and predominantly negative relations of HOMA-IR to LPCs and SMs (see S4 Table).

Insulin resistance is often accompanied with changes in desaturase activities In the present study, D5D tended to decrease from NGT to T2D individuals while D6D and D9D were increased significantly in IGT and T2D subjects (Table 3). All desaturase activities showed significant associations to three or more insulin sensitivity indices Table 4.

**Table 3:** Desaturase activities.

A)	Enzyme activity			p-value		
	NGT	IGT	T2D	NGT v. IGT	NGT v. T2D	IGT v. T2D
**D5D**	22.94	19.41	17.50	NS	NS	NS
**D6D**	0.364	0.523	0.487	0.001	0.002	NS
**D9D**	0.367	0.593	0.534	0.006	0.002	NS

**Table 4:** Correlation coefficients of desaturase activities to insulin sensitivity indices.

B)	Spearman rho correlation coefficients to sensitivity indices (significance)			
	HOMA-IR	GSI	ISI	DI
**D5D**	-0.268	0.267	0.209	0.080
	(0.011)	(0.011)	(0.050)	(0.455)
**D6D**	0.244	-0.162	-0.426	-0.374
	(0.021)	(0.127)	(0.000)	(0.000)
**D9D**	0.273	-0.223	-0.373	-0.297
	(0.010)	(0.035)	(0.000)	(0.005)

To identify a lipidomic signature of each investigated insulin sensitivity index, we applied LASSO selection procedure. This model considered all measured lipid species simultaneously; age, BMI, WHR, and systolic blood pressure were included in the model. After LASSO selection, the plasma lipidome contributed to 3% variability in DI, 45% variability in GSI, 52% variability in ISI, and to maximal 53% variability in HOMA-IR which was calculated using R-Squared statistics.

For the four investigated insulin sensitivity indexes LASSO selected lipid species from 8 lipid classes: most represented were TAG species (10), followed by DAGs (3), LPCs (3), SMs (3), LPEs (3), PCs (2), PC O-s (1), and PE-O-s (1). Of note, only four lipid species were selected two or three times for different indices: DAG 38:5 –HOMA-IR, GSI and ISI, LPE 22:6 -HOMA-IR and GSI, TAG 46:0 -HOMA-IR and ISI, TAG 46:1 -HOMA-IR and DI, suggesting that lipid metabolism is heterogeneously involved in mechanistic aspects of insulin resistance.

**Fig 3:**
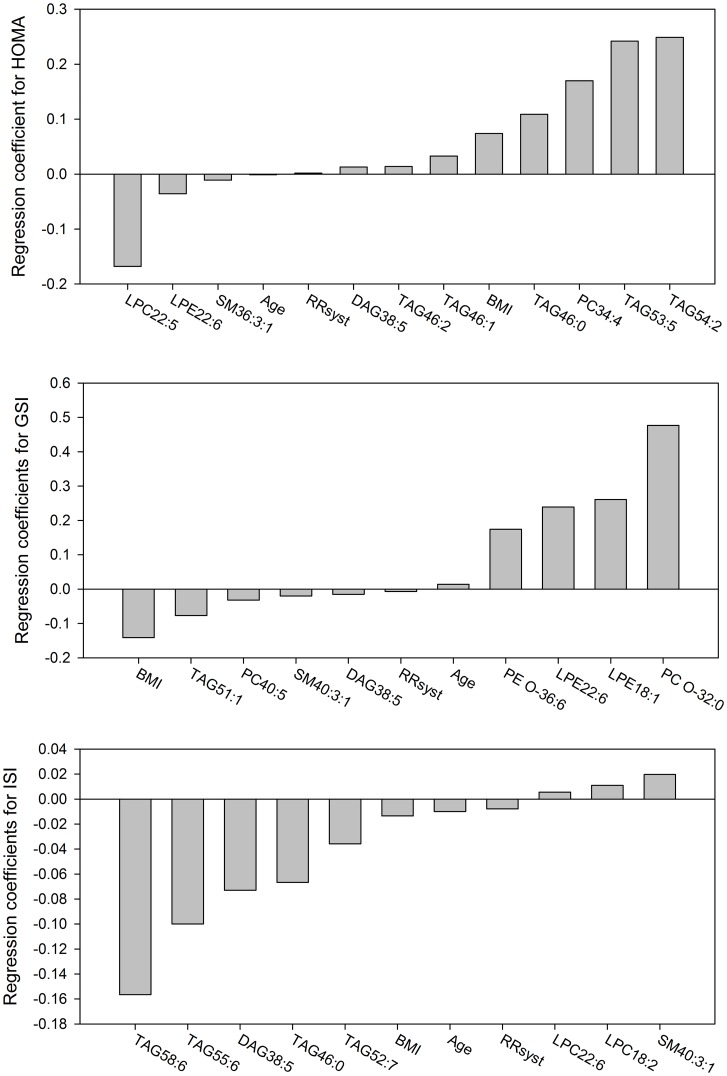
Strength and direction of LASSO regression coefficients for the insulin sensitivity indices HOMA-IR, GSI, and ISI.

Fig 3 shows the regression coefficients of selected lipid species and anthropometric values for HOMA-IR, GSI, and ISI. For HOMA-IR ten distinct lipid species were selected, the highest positive regression coefficient was obtained for TAG 54:2, the highest negative regression coefficient for LPC 22:5. For GSI eight molecular species were selected including PC O- 32:0, highest positive regression coefficient, and TAG 51:1, highest negative regression coefficient. LASSO procedure selected eight species for ISI with SM 40:3:1 having highest positive regression coefficient and TAG 58:6 having highest negative regression coefficient. LASSO selected only one lipid species for DI—TAG 46:1, regression coefficient: -0.097. Age, systolic blood pressure and BMI were selected for HOMA-IR, GSI, and ISI, while BMI and systolic blood pressure were selected for DI.

## Discussion

Lipid abnormalities have been shown to be causally involved in the pathogenesis of T2D and its cardiovascular complications. In this respect, the key concept of glucolipotoxicity is increasingly recognized as a potential molecular mechanism mediating insulin resistance and its clinical consequences [20, 21]. Up to now, clinicians traditionally operate with integrated indices of lipid metabolism including circulating free fatty acids, total cholesterol, and triglycerides. Accurate plasma lipidomic profiling, however, suggests that the interaction between lipid composition and disease is subtle and might contribute to disease risk prediction and therapy monitoring [8, 22–24].

Male subjects investigated in our study, showed a broad range of insulin sensitivity, that was identified by four distinct surrogate markers. HOMA-IR is a simple but effective measure of insulin resistance in fasting steady state conditions while ISI is a more complex estimation of insulin sensitivity incorporating glucose and insulin levels at the beginning (0 min) and end (120 min) of OGTT and body weight. ISI and GSI have been shown to correlate well with the insulin sensitivity index obtained from the euglycemic hyperinsulinemic clamp, the gold standard for measurement of insulin resistance [12, 25]. DI has been shown to be a valuable surrogate measure of ß-cell function reflecting the ability of the ß-cell to compensate for insulin resistance. While the static sensitivity index HOMA-IR mainly reflects hepatic insulin sensitivity, dynamic indices, including both fasting and stimulated glucose and insulin levels, reflect hepatic as well peripheral insulin sensitivity [26]. Consequently, different surrogate indices capture different pathophysiological aspects of glucose intolerance [27, 28]. These differences might be an explanation for the fact that LASSO selection procedure included distinct individual lipid species for each investigated insulin sensitivity index. Only DAG 38:5 was included in three of four sensitivity indexes models: HOMA-IR (positive regression coefficient), GSI and ISI (negative regression coefficients). Accumulating evidence suggests that lipid-induced insulin resistance is at least partially mediated by diacylglycerols that activate novel protein kinase C isoforms with subsequent inhibition of insulin action in liver and skeletal muscle [29, 30]. Interestingly, DAG species, specifically DAG 16:0/22:5 and DAG 16:0/22:6, were also associated with increased blood pressure and the liability of incident hypertension, an integral part of the metabolic syndrome [31].

Hypertriglyceridemia is an independent risk factor of T2D. In support of this, the present study demonstrates a close association between TAG species and insulin sensitivity indices, particularly HOMA-IR and ISI. We observed a signature in which TAGs with lower carbon number and double bond content were associated with higher positive correlation to HOMA-IR and negative association to ISI. A similar lipid pattern of TAG and HOMA-IR association was also reported previously [32]. Rhee et al. [32] identified 6 TAG species that were associated with increased risk of diabetes and 3 TAGs that were related to decreased risk of T2D. In the present study, TAGs were among the most frequently selected lipid species after LASSO procedure, especially for HOMA-IR (five positive regression coefficients—TAG 54:2, TAG 53:5, TAG 46:0, TAG 46:1, TAG 46:2) and for ISI (four negative regression coefficients—TAG 58:6, TAG 55:6, TAG 46:0, TAG 52:7) thus emphasizing the pathophysiological importance of specific TAGs for insulin resistance. In conclusion, TAG and DAG plasma profiling provides a more differentiated view on changes in lipid homeostasis of individuals with glucose intolerance and, moreover, may improve diabetes risk prediction [33, 34].

Insulin resistance is characterized by specific changes in the fatty acid profile in plasma and skeletal muscle membranes including increased palmitic (FA 16:0) and decreased linoleic (FA 18:2 *n-6*) acid [35, 36]. One mechanism for the shifted fatty acid pattern may include activation of Δ6 and Δ9 desaturase by insulin [37]. Typically, insulin resistance is associated with an increase of D6D and D9D activity as well as a decrease in D5D activity [36]. Similar results were obtained in the present study. Consequently, D6D and D9D were significantly positively related to HOMA-IR, while D5D was negatively associated to HOMA-IR (Table 4).

SMs are structural components of tissue membranes, important cellular messengers, and are abundant in nerve cells. In the present study, a substantial part of SM molecular species showed a strong negative relation to HOMA-IR and a significant positive association to ISI and DI suggesting a positive role for SMs in insulin sensitivity. Accordingly, SM 40:3:1 had the highest positive regression coefficient for ISI after LASSO selection. Our findings confirm previous investigations showing that that several SM species were downregulated in diabetes [33]. In contrast, Hanamatsu et al. [38] reported a positive correlation of several SM species in serum with HOMA-IR in obese individuals.

Ether phosphatidylcholines (PC O-) are suggested to have antioxidant and cardioprotective properties. Downregulation of PC O-s has been reported in hypertension [8] and Crohn’s disease [39]. In addition, higher levels of PC O-s were associated with familial longevity [40]. Only sparse previous studies document an involvement of ether lipids in the pathogenesis of T2D. Pietilainen et al. [41] found that PC O-s were associated with better insulin sensitivity. In a targeted lipidomics study of a prediabetic pig model Renner et al. report a decrease of several circulating PC O- species with time [42]. In support of this, our study documents the highest positive regression coefficient of PC O- 32:0 for GSI. However, PC O-s did not play a significant positive or negative role for the other insulin sensitivity indices HOMA-IR, ISI, and DI.

In summary, using sensitive shotgun lipidomics, we demonstrate in the present study varying relationships of plasma lipid species to four distinct insulin sensitivity indices. After LASSO selection the plasma lipidome explained up to 53% variability of the sensitivity indices. Close association of insulin sensitivity indices with a large number of molecular lipid species reflects the importance of changes in lipid homeostasis in the pathogenesis of T2D.

## Supporting Information

S1 Fig Spearman rank correlation coefficients of insulin senitivity/resistance indices with different lipid classes; black bars indicate statistically significant correlations: *p<0.05, **p<0.01, ***p<0.001, white bars: not significant. (TIFF)

S2 Fig Heat map showing the logarthmic values of lipid classes in the plasma of the study group according to their status of glucose tolerance (green = NGT, yellow = IGT, red = T2D). (TIFF)

S1 Table (DOC)

S2 Table Internal standard used for lipidomics of blood plasma lipids. Names of lipid classes were abbreviated as annotated.
(DOCX)

S3 Table Statistical test results revealing distinct plasma concentrations of lipid species in NGT, IGT, and T2D individuals. Overall tests for differential concentrations was conducted by ANOVA, pairwise comparisons by t-tests.
(DOCX)

S4 Table Correlations of lipid species with different insulin sensitivity indices. (DOCX)
