# Supplementary figures and images for: Detection of Independent Associations of Plasma Lipidomic Parameters with Insulin Sensitivity Indices Using Data Mining Methodology
Source: PLoS One. 2016 Oct 13;11(10):e0164173. doi: 10.1371/journal.pone.0164173 (PMC5063331; doi:10.1371/journal.pone.0164173)

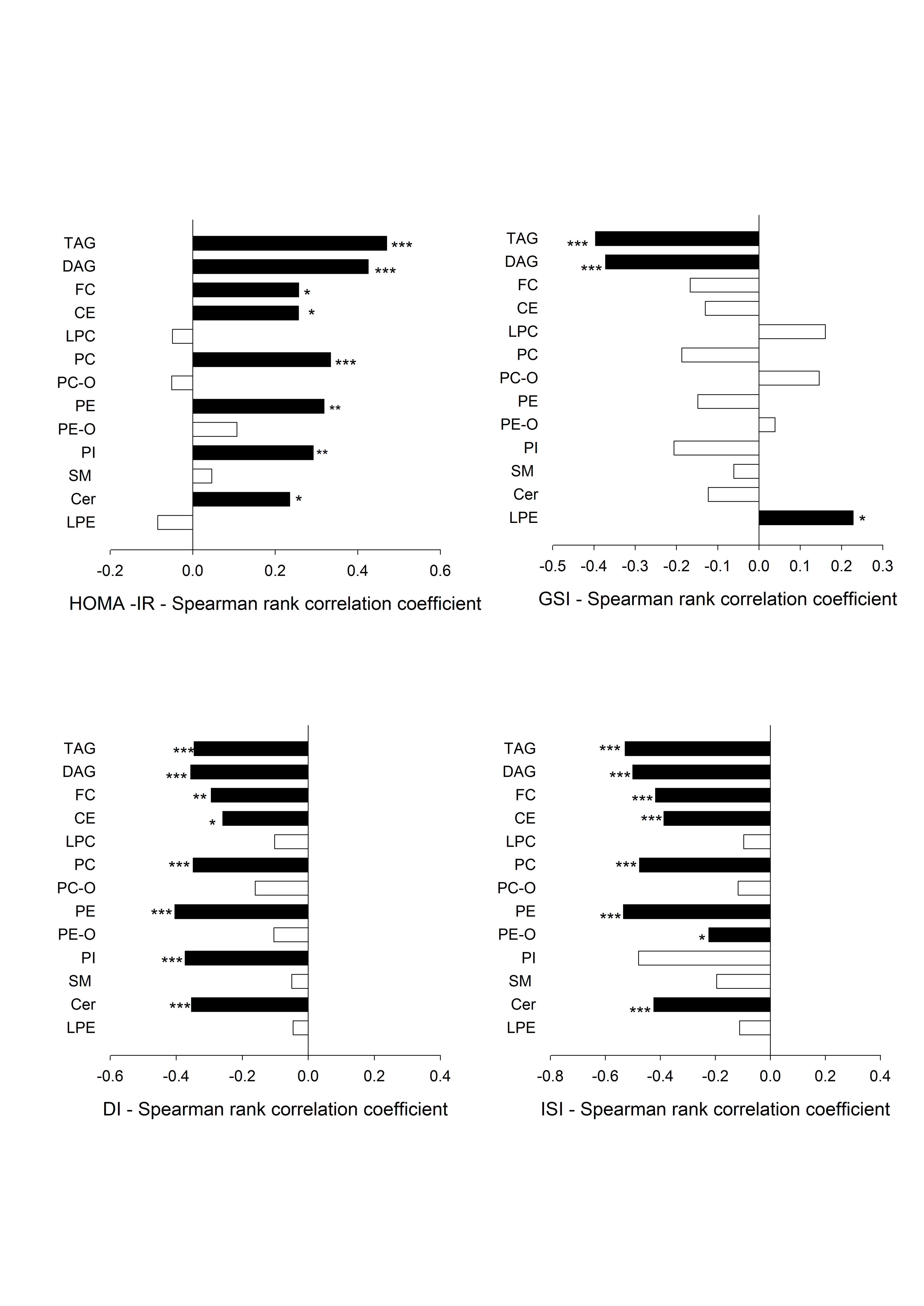

Supplement: S1 Fig — (TIFF) [file pone.0164173.s001.tiff]

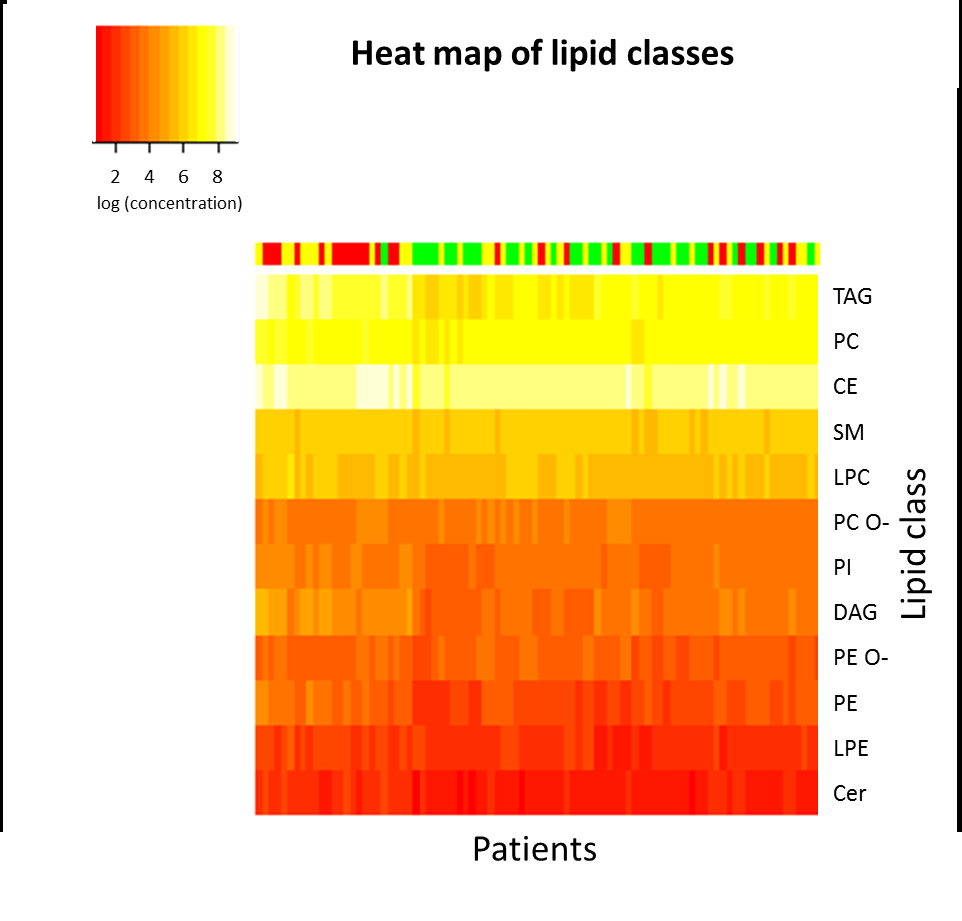

Supplement: S2 Fig — (TIFF) [file pone.0164173.s002.tiff]
