# Supplementary material for: Detection of Independent Associations of Plasma Lipidomic Parameters with Insulin Sensitivity Indices Using Data Mining Methodology
Source: PLoS One. 2016 Oct 13;11(10):e0164173. doi: 10.1371/journal.pone.0164173 (PMC5063331; doi:10.1371/journal.pone.0164173)
Supplement: S1 Table — (DOC) [file pone.0164173.s003.doc]

Supplemental Table 1

Internal standard used for lipidomics of blood plasma lipids. Names of lipid classes were abbreviated as annotated.

| Internal standard | Amount used for extraction, nmol | Quantified lipid class | |
| --- | --- | --- | --- |
|  |  | Name | Abbreviation |
| CE 12:0 | 5.63 | Cholesterol ester | CE |
| CholD7 | 4.15 | Cholesterol | Chol |
| TAG 36:0 | 1.43 | Triacylglycerol | TAG |
| TAGD5 52:0 | 0.28 |  |  |
| DAG 24:0 | 0.30 | Diacylglycerol | DAG |
| DAGD5 34:0 | 0.14 |  |  |
| PC 25:0 | 1.47 | Phosphatidylcholine | PC |
| PC 43:6 | 0.05 | Phosphatidylcholine plasmalogen/ether | PC O- |
| LPC 13:0 | 0.58 | Lysophosphatidylcholine | LPC |
| PE 25:0 | 0.26 | Phosphatidylethanolamine | PE |
|  |  | Phosphatidylethanolamine plasmalogen/ether | PE O- |
| LPE 13:0 | 0.26 | Lysophosphatidylethanolamine | LPE |
| PI 25:0 | 0.21 | Phosphatidylinositol | PI |
| PI 43:6 | 0.04 |  |  |
| SM 30:1:1 | 0.71 | Sphingomyelin | SM |
| Cer 30:1:2 | 0.16 | Ceramide | Cer |
