# Supplementary material for: Detection of Independent Associations of Plasma Lipidomic Parameters with Insulin Sensitivity Indices Using Data Mining Methodology
Source: PLoS One. 2016 Oct 13;11(10):e0164173. doi: 10.1371/journal.pone.0164173 (PMC5063331; doi:10.1371/journal.pone.0164173)
Supplement: S2 Table — Names of lipid classes were abbreviated as annotated. (DOCX) [file pone.0164173.s004.docx]

Supplemental Table 2

Statistical test results revealing distinct plasma concentrations of lipid species in NGT, IGT, and T2D individuals

Page 1

| Parameter  (µmol/l) | NGT  Mean (SD) | IGT  Mean (SD) | T2D  Mean (SD) | F-Statistic | p-value | IGT vs. NGT  (T-Statistic) | IGT vs. NGT  (p-value) | IGT vs. T2D  (T-Statistic) | IGT vs. T2D  (p-value) | NGT vs. T2D  (T-Statistic) | NGT vs. T2D  (p-value) |
| --- | --- | --- | --- | --- | --- | --- | --- | --- | --- | --- | --- |
| TAG44:0 | -0.486 (0.833) | 0.043 (1.050) | 0.449 (0.961) | 7.067 | 0.007 | 12.612 | <0.001 | 14.698 | <0.001 | 10.490 | <0.001 |
| TAG46:0 | 0.612 (0.786) | 1.185 (0.920) | 1.614 (0.815) | 10.307 | 0.001 | 4.690 | <0.001 | 7.850 | <0.001 | 4.549 | <0.001 |
| TAG46:1 | 1.070 (1.067) | 1.681 (0.993) | 2.132 (0.824) | 8.634 | 0.003 | 0.832 | NS | 3.965 | <0.001 | 1.717 | <0.05 |
| TAG46:2 | 0.772 (0.724) | 1.166  (0.800) | 1.456 (0.720) | 6.068 | 0.012 | 4.562 | <0.001 | 9.394 | <0.001 | 4.749 | <0.001 |
| TAG47:1 | -0.088 (0.688) | 0.182 (0.795) | 0.462 (0.623) | 4.277 | 0.041 | 12.924 | <0.001 | 18.158 | <0.001 | 10.642 | <0.001 |
| TAG47:2 | -0.605 (0.610) | -0.382 (0.793) | -0.180 (0.609) | 2.807 | 0.119 | 18.413 | <0.001 | 23.539 | <0.001 | 14.657 | <0.001 |
| TAG48:1 | 2.815 (0.667) | 3.321 (0.752) | 3.624 (0.632) | 10.322 | 0.001 | -14.036 | <0.001 | -8.807 | <0.001 | -7.887 | <0.001 |
| TAG48:2 | 2.686 (0.572) | 3.063 (0.684) | 3.302 (0.587) | 7.399 | 0.006 | -13.487 | <0.001 | -6.717 | <0.001 | -6.958 | <0.001 |
| TAG48:3 | 1.722 (0.477) | 1.955 (0.567) | 2.167 (0.473) | 5.500 | 0.017 | -3.789 | <0.001 | 4.025 | <0.001 | -0.350 | NS |
| TAG49:1 | 1.152 (0.536) | 1.394 (0.575) | 1.680 (0.555) | 6.436 | 0.010 | 2.354 | <0.05 | 9.064 | <0.001 | 3.150 | <0.01 |
| TAG49:2 | 1.092 (0.425) | 1.311 (0.526) | 1.473 (0.461) | 4.767 | 0.028 | 3.369 | 0.001 | 11.263 | <0.001 | 4.168 | <0.001 |
| TAG49:3 | 0.180 (0.315) | 0.333 (0.462) | 0.478 (0.414) | 4.000 | 0.050 | 15.518 | <0.001 | 23.037 | <0.001 | 11.041 | <0.001 |
| TAG50:1 | 3.788 (0.544) | 4.248 (0.674) | 4.577 (0.589) | 12.513 | 0.000 | -24.741 | <0.001 | -17.919 | <0.001 | -14.252 | <0.001 |

Supplemental Table 2

Statistical test results revealing distinct concentrations of lipid species in NGT, IGT, and T2D individuals

Page 2

| Parameter  (µmol/l) | NGT  Mean (SD) | IGT  Mean (SD) | T2D  Mean (SD) | F-Statistic | p-value | IGT vs. NGT  (T-Statistic) | IGT vs. NGT  (p-value) | IGT vs. T2D  (T-Statistic) | IGT vs. T2D  (p-value) | NGT vs. T2D  (T-Statistic) | NGT vs. T2D  (p-value) |
| --- | --- | --- | --- | --- | --- | --- | --- | --- | --- | --- | --- |
| TAG50:2 | 4.380 (0.420) | 4.740 (0.565) | 4.922 (0.466) | 9.467 | 0.002 | -33.957 | <0.001 | -24.736 | <0.001 | -18.652 | <0.001 |
| TAG50:3 | 3.652 (0.343) | 3.918 (0.432) | 4.016 (0.416) | 6.768 | 0.008 | -28.473 | <0.001 | -17.520 | <0.001 | -13.707 | <0.001 |
| TAG50:4 | 2.430 (0.329) | 2.562 (0.403) | 2.741 (0.385) | 4.957 | 0.025 | -12.922 | <0.001 | -2.371 | <0.05 | -4.992 | <0.001 |
| TAG50:5 | 0.898 (0.389) | 1.089 (0.447) | 1.206 (0.408) | 4.113 | 0.046 | 6.109 | <0.001 | 14.835 | <0.001 | 5.835 | <0.001 |
| TAG51:1 | 0.396 (0.562) | 0.694 (0.580) | 0.931 (0.641) | 5.952 | 0.013 | 9.904 | <0.001 | 15.643 | <0.001 | 7.896 | <0.001 |
| TAG51:2 | 2.047 (0.364) | 2.240 (0.422) | 2.406 (0.419) | 5.809 | 0.014 | -8.125 | <0.001 | 1.444 | NS | -2.384 | <0.05 |
| TAG51:3 | 1.697 (0.283) | 1.745 (0.365) | 1.891 (0.407) | 2.283 | 0.182 | -3.073 | <0.01 | 7.508 | <0.001 | 0.587 | NS |
| TAG51:4 | 0.689 (0.296) | 0.681 (0.378) | 0.812 (0.396) | 1.147 | 0.447 | 10.741 | <0.001 | 20.257 | <0.001 | 8.071 | <0.001 |
| TAG51:5 | -371 (0.291) | -0.161 (0.437) | -0.012 (0.420) | 6.416 | 0.010 | 22.441 | <0.001 | 28.927 | <0.001 | 14.748 | <0.001 |
| TAG52:2 | 5.432 (0.446) | 5.628 (0.416) | 5.783 (0.414) | 4.932 | 0.026 | -48.664 | <0.001 | -37.688 | <0.001 | -25.730 | <0.001 |
| TAG52:3 | 5.386 (0.327) | 5.472 (0.326) | 5.559 (0.403) | 1.766 | 0.268 | -52.840 | <0.001 | -37.577 | <0.001 | -25.738 | <0.001 |
| TAG52:4 | 4.590 (0.394) | 4.540 (0.347) | 4.688 (0.398) | 1.090 | 0.463 | -39.653 | <0.001 | -26.172 | <0.001 | -19.636 | <0.001 |
| TAG52:5 | 3.175 (0.336) | 3.218 (0.318) | 3.320 (0.412) | 1.236 | 0.416 | -22.898 | <0.001 | -10.061 | <0.001 | -9.817 | <0.001 |

Supplemental Table 2

Statistical test results revealing distinct concentrations of lipid species in NGT, IGT, and T2D individuals

Page 3

| Parameter  (µmol/l) | NGT  Mean (SD) | IGT  Mean (SD) | T2D  Mean (SD) | F-Statistic | p-value | IGT vs. NGT  (T-Statistic) | IGT vs. NGT  (p-value) | IGT vs. T2D  (T-Statistic) | IGT vs. T2D  (p-value) | NGT vs. T2D  (T-Statistic) | NGT vs. T2D  (p-value) |
| --- | --- | --- | --- | --- | --- | --- | --- | --- | --- | --- | --- |
| TAG52:6 | 1.688 (0.359) | 1.869 (0.422) | 1.964 (0.464) | 3.400 | 0.076 | -3.579 | 0.001 | 5.987 | <0.001 | 0.386 | NS |
| TAG52:7 | -0.043 (0.474) | 0.233 (0.584) | 0.318 (0.593) | 3.593 | 0.068 | 15.202 | <0.001 | 21.275 | <0.001 | 11.665 | <0.001 |
| TAG53:2 | 1.413 (0.317) | 1.495 (0.389) | 1.599 (0.399) | 1.836 | 0.255 | 0.513 | NS | 10.652 | <0.001 | 2.644 | <0.05 |
| TAG53:3 | 1.728 (0.258) | 1.705 (0.350) | 1.801 (0.400) | 0.610 | 0.660 | -3.071 | <0.01 | 8.371 | <0.001 | 0.744 | NS |
| TAG53:4 | 1.187 (0.256) | 1.154 (0.355) | 1.253 (0.392) | 0.625 | 0.654 | 4.395 | <0.001 | 15.028 | <0.001 | 4.695 | <0.001 |
| TAG53:5 | 0.219 (0.354) | 0.212 (0.373) | 0.346 (0.389) | 1.116 | 0.454 | 16.612 | <0.001 | 25.98 | <0.001 | 11.350 | <0.001 |
| TAG53:6 | 0.530 (0.355) | 0.785 (0.422) | 0.873 (0.359) | 6.584 | 0.009 | 10.413 | <0.001 | 19.127 | <0.001 | 8.401 | <0.001 |
| TAG53:7 | 0.432 (0.490) | 0.602 (0.440) | 0.705 (0.433) | 2.661 | 0.133 | 11.421 | <0.001 | 20.371 | <0.001 | 9.008 | <0.001 |
| TAG54:1 | 0.563 (0.481) | 0.552 (0.435) | 0.633 (0.426) | 0.260 | 0.826 | 11.096 | <0.001 | 21.193 | <0.001 | 8.801 | <0.001 |
| TAG54:2 | 2.102 (0.631) | 2.308 (0.559) | 2.586 (0.491) | 5.136 | 0.022 | -7.311 | <0.001 | 0.086 | NS | -2.908 | <0.01 |
| TAG54:3 | 4.099 (0.375) | 4.079 (0.403) | 4.182 (0.383) | 0.536 | 0.678 | -32.979 | <0.001 | -19.973 | <0.001 | -16.205 | <0.001 |
| TAG54:4 | 4.108 (0.409) | 4.009 (0.386) | 4.087 (0.407) | 0.535 | 0.678 | -32.229 | <0.001 | -19.019 | <0.001 | -15.803 | <0.001 |
| TAG54:5 | 3.792 (0.392) | 3.724 (0.283) | 3.841 (0.339) | 0.843 | 0.566 | -30.004 | <0.001 | -17.116 | <0.001 | -13.956 | <0.001 |

Supplemental Table 2

Statistical test results revealing distinct concentrations of lipid species in NGT, IGT, and T2D individuals

Page 4

| Parameter  (µmol/l) | NGT  Mean (SD) | IGT  Mean (SD) | T2D  Mean (SD) | F-Statistic | p-value | IGT vs. NGT  (T-Statistic) | IGT vs. NGT  (p-value) | IGT vs. T2D  (T-Statistic) | IGT vs. T2D  (p-value) | NGT vs. T2D  (T-Statistic) | NGT vs. T2D  (p-value) |
| --- | --- | --- | --- | --- | --- | --- | --- | --- | --- | --- | --- |
| TAG54:6 | 3.302 (0.339) | 3.349 (0.299) | 3.415 (0.373) | 0.802 | 0.578 | -24.806 | <0.001 | -11.810 | <0.001 | -10.694 | <0.001 |
| TAG54:7 | 2.329 (0.426) | 2.404 (0.461) | 2.495 (0.503) | 0.918 | 0.533 | -10.510 | <0.001 | -0.056 | NS | -3.722 | <0.001 |
| TAG54:8 | 0.696 (0.475) | 0.781 (0.613) | 0.866 (0.678) | 0.600 | 0.662 | 8.199 | <0.001 | 15.089 | <0.001 | 7.226 | <0.001 |
| TAG55:6 | -0.307 (0.473) | -0.018 (0.416) | 0.042 (0.430) | 5.466 | 0.017 | 19.474 | <0.001 | 28.099 | <0.001 | 13.850 | <0.001 |
| TAG55:7 | -0.196 (0.410) | 0.031 (0.452) | 0.087 (0.500) | 3.345 | 0.079 | 18.948 | <0.001 | 26.196 | <0.001 | 13.360 | <0.001 |
| TAG56:3 | 0.364 (0.623) | 0.384 (0.596) | 0.553 (0.606) | 0.785 | 0.584 | 11.447 | <0.001 | 19.115 | <0.001 | 9.186 | <0.001 |
| TAG56:4 | 0.618 (0.572) | 0.633 (0.382) | 0.728 (0.387) | 0.456 | 0.723 | 10.003 | <0.001 | 21.106 | <0.001 | 8.158 | <0.001 |
| TAG56:5 | 2.144 (0.380) | 2.200 (0.247) | 2.236 (0.379) | 0.549 | 0.678 | -9.178 | <0.001 | 2.856 | <0.01 | -2.295 | <0.05 |
| TAG56:6 | 2.836 (0.299) | 2.959 (0.249) | 2.927 (0.342) | 1.507 | 0.336 | -19.630 | <0.001 | -6.601 | <0.001 | -7.357 | <0.001 |
| TAG56:7 | 3.151 (0.365) | 3.295 (0.409) | 3.373 (0.481) | 2.155 | 0.199 | -21.859 | <0.001 | -10.086 | <0.001 | -9.696 | <0.001 |
| TAG56:8 | 2.501 (0.467) | 2.531 (0.521) | 2.591 (0.566) | 0.222 | 0.854 | -11.734 | <0.001 | -1.225 | NS | -4.605 | <0.001 |
| TAG56:9 | 1.000  (0.508) | 1.068 (0.625) | 1.139 (0.691) | 0.376 | 0.756 | 4.882 | <0.001 | 12.326 | <0.001 | 5.259 | <0.001 |
| TAG58:6 | -0.044 (0.536) | 0.266 (0.393) | 0.431 (0.395) | 8.450 | 0.003 | 15.828 | <0.001 | 24.762 | <0.001 | 11.447 | <0.001 |

Supplemental Table 2

Statistical test results revealing distinct concentrations of lipid species in NGT, IGT, and T2D individuals

Page 5

| Parameter  (µmol/l) | NGT  Mean (SD) | IGT  Mean (SD) | T2D  Mean (SD) | F-Statistic | p-value | IGT vs. NGT  (T-Statistic) | IGT vs. NGT  (p-value) | IGT vs. T2D  (T-Statistic) | IGT vs. T2D  (p-value) | NGT vs. T2D  (T-Statistic) | NGT vs. T2D  (p-value) |
| --- | --- | --- | --- | --- | --- | --- | --- | --- | --- | --- | --- |
| TAG58:7 | 1.069 (0.333) | 1.261 (0.335) | 1.370 (0.350) | 5.988 | 0.013 | 4.347 | <0.001 | 14.063 | <0.001 | 4.744 | <0.001 |
| TAG58:8 | 1.665 (0.408) | 1.734 (0.503) | 1.763 (0.506) | 0.345 | 0.763 | -2.452 | <0.05 | 7.380 | <0.001 | 1.076 | NS |
| TAG58:9 | 1.385 (0.466) | 1.403 (0.532) | 1.433 (0.546) | 0.064 | 0.943 | 1.125 | NS | 10.553 | <0.001 | 3.114 | <0.01 |
| TAG58:10 | 1.016 (0.407) | 1.043 (0.542) | 1.082 (0.542) | 0.126 | 0.910 | 5.390 | <0.001 | 14.166 | <0.001 | 5.644 | <0.001 |
| CE14:0 | 3.339 (0.359) | 3.428 (0.285) | 3.450 (0.330) | 0.992 | 0.500 | -25.412 | <0.001 | -12.891 | <0.001 | -10.972 | <0.001 |
| CE15:0 | 2.106 (0.349) | 1.991 (0.348) | 1.934 (0.411) | 1.681 | 0.287 | -7.316 | <0.001 | 5.705 | <0.001 | -1.207 | NS |
| CE16:0 | 5.895 (0.157) | 5.829 (0.247) | 5.801 (0.240) | 1.501 | 0.336 | -64.662 | <0.001 | -45.858 | <0.001 | -29.840 | <0.001 |
| CE16:1 | 4.920 (0.262) | 5.186 (0.440) | 5.130 (0.417) | 4.463 | 0.036 | -45.373 | <0.001 | -31.244 | <0.001 | -22.646 | <0.001 |
| CE17:1 | 2.431 (0.248) | 2.441 (0.250) | 2.403 (0.326) | 0.148 | 0.901 | -13.562 | <0.001 | 0.186 | NS | -4.085 | <0.001 |
| CE18:1 | 6.389 (0.150) | 6.404 (0.147) | 6.404 (0.225) | 0.077 | 0.938 | -75.313 | <0.001 | -56.132 | <0.001 | -33.981 | <0.001 |
| CE18:2 | 7.686 (0.203) | 7.686  (0.188) | 7.772  (0.228) | 1.607 | 0.306 | 92.483 | <0.001 | -73.546 | <0.001 | -43.661 | <0.001 |
| CE18:3 | 4.524 (0.295) | 4.581 (0.268) | 4.497 (0.374) | 0.562 | 0.675 | -42.753 | <0.001 | -26.788 | <0.001 | -19.191 | <0.001 |
| CE19:2 | 2.364 (0.345) | 2.127 (0.399) | 1.894 (0.363) | 11.598 | 0.001 | -9.572 | <0.001 | 4.886 | <0.001 | -2.105 | <0.05 |

Supplemental Table 2

Statistical test results revealing distinct concentrations of lipid species in NGT, IGT, and T2D individuals

Page 6

| Parameter  (µmol/l) | NGT  Mean (SD) | IGT  Mean (SD) | T2D  Mean (SD) | F-Statistic | p-value | IGT vs. NGT  (T-Statistic) | IGT vs. NGT  (p-value) | IGT vs. T2D  (T-Statistic) | IGT vs. T2D  (p-value) | NGT vs. T2D  (T-Statistic) | NGT vs. T2D  (p-value) |
| --- | --- | --- | --- | --- | --- | --- | --- | --- | --- | --- | --- |
| CE20:3 | 2.994 (0.328) | 3.022 (0.345) | 3.077 (0.395) | 0.395 | 0.747 | -20.209 | <0.001 | -7.399 | <0.001 | -8.378 | <0.001 |
| CE20:4 | 5.970 (0.290) | 5.870 (0.387) | 5.789 (0.294) | 2.190 | 0.195 | -58.593 | <0.001 | -42.008 | <0.001 | -29.396 | <0.001 |
| CE20:5 | 4.559 (0.461) | 4.563 (0.600) | 4.425 (0.624) | 0.534 | 0.678 | -33.848 | <0.001 | -19.763 | <0.001 | -17.721 | <0.001 |
| CE22:6 | 3.719 (0.408) | 3.542 (0.544) | 3.499 (0.448) | 1.864 | 0.250 | -24.682 | <0.001 | -11.569 | <0.001 | -12.315 | <0.001 |
| Cer40:1:2 | -0.340 (0.380) | -0.304 (0.381) | -0.218 (0.252) | 0.886 | 0.546 | 23.228 | <0.001 | 34.031 | <0.001 | 15.543 | <0.001 |
| Cer41:1:2 | -0.361 (0.411) | -0.349 (0.368) | -0.214 (0.346) | 1.259 | 0.410 | 23.429 | <0.001 | 33.352 | <0.001 | 15.345 | <0.001 |
| Cer42:1:2 | 0.935 (0.256) | 0.883 (0.274) | 0.938 (0.254) | 0.426 | 0.733 | 8.261 | <0.001 | 20.419 | <0.001 | 6.841 | <0.001 |
| Cer42:2:2 | 0.503 (0.244) | 0.495 (0.239) | 0.527 (0.286) | 0.113 | 0.917 | 14.347 | <0.001 | 25.852 | <0.001 | 9.948 | <0.001 |
| LPE16:0 | 0.167 (0.309) | 0.085 (0.347) | -0.079 (0.406) | 3.533 | 0.069 | 18.309 | <0.001 | 29.144 | <0.001 | 12.867 | <0.001 |
| LPE18:0 | 0.920 (0.192) | 0.859 (0.257) | 0.770 (0.150) | 3.676 | 0.064 | 8.794 | <0.001 | 22.362 | <0.001 | 7.541 | <0.001 |
| LPE18:1 | 0.243 (0.425) | 0.043 (0.374) | -0.144 (0.404) | 6.638 | 0.009 | 16.724 | <0.001 | 29.326 | <0.001 | 12.387 | <0.001 |
| LPE18:2 | 0.857 (0.461) | 0.571 (0.368) | 0.402 (0.418) | 8.889 | 0.003 | 9.367 | <0.001 | 22.963 | <0.001 | 8.207 | <0.001 |
| LPE20:4 | 0.463 (0.416) | 0.408 (0.362) | 0.202 (0.300) | 3.811 | 0.058 | 13.386 | <0.001 | 26.232 | <0.001 | 10.734 | <0.001 |

Supplemental Table 2

Statistical test results revealing distinct concentrations of lipid species in NGT, IGT, and T2D individuals

Page 7

| Parameter  (µmol/l) | NGT  Mean (SD) | IGT  Mean (SD) | T2D  Mean (SD) | F-Statistic | p-value | IGT vs. NGT  (T-Statistic) | IGT vs. NGT  (p-value) | IGT vs. T2D  (T-Statistic) | IGT vs. T2D  (p-value) | NGT vs. T2D  (T-Statistic) | NGT vs. T2D  (p-value) |
| --- | --- | --- | --- | --- | --- | --- | --- | --- | --- | --- | --- |
| LPE22:6 | 0.438 (0.390) | 0.307 (0.560) | 0.184 (0.396) | 2.199 | 0.195 | 12.935 | <0.001 | 23.448 | <0.001 | 10.818 | <0.001 |
| LPC14:0 | 0.646 (0.361) | 0.693 (0.341) | 0.641 (0.305) | 0.216 | 0.854 | 10.824 | <0.001 | 22.387 | <0.001 | 8.802 | <0.001 |
| LPC16:0 | 5.141 (0.173) | 4.977 (0.280) | 4.874 (0.223) | 10.036 | 0.001 | -51.360 | <0.001 | -33.383 | <0.001 | -23.473 | <0.001 |
| LPC16:1 | 1.188 (0.278) | 1.324 (0.420) | 1.122 (0.315) | 2.610 | 0.138 | 3.091 | <0.01 | 14.345 | <0.001 | 5.138 | <0.001 |
| LPC17:0 | 0.537 (0.391) | 0.194 (0.707) | 0.139 (0.458) | 4.895 | 0.026 | 11.673 | <0.001 | 21.781 | <0.001 | 10.352 | <0.001 |
| LPC18:0 | 3.868 (0.225) | 3.712 (0.360) | 3.590 (0.240) | 6.967 | 0.007 | -31.417 | <0.001 | -15.554 | <0.001 | -13.887 | <0.001 |
| LPC18:1 | 3.529 (0.288) | 3.358 (0.361) | 3.138 (0.321) | 10.316 | 0.001 | -26.019 | <0.001 | -10.085 | <0.001 | -10.751 | <0.001 |
| LPC18:2 | 3.909 (0.414) | 3.476 (0.452) | 3.257 (0.450) | 17.015 | 0.000 | -25.489 | <0.001 | -10.453 | <0.001 | -11.862 | <0.001 |
| LPC18:3 | -0.388 (0.704) | -0.476 (0.447) | -0.539 (0.658) | 0.450 | 0.723 | 20.023 | <0.001 | 30.019 | <0.001 | 14.572 | <0.001 |
| LPC20:3 | 1.085 (0.333) | 1.001 (0.350) | 0.883 (0.314) | 2.601 | 0.138 | 5.942 | <0.001 | 18.630 | <0.001 | 6.238 | <0.001 |
| LPC20:4 | 2.445 (0.391) | 2.294 (0.436) | 2.086 (0.340) | 5.879 | 0.013 | -10.821 | <0.001 | 2.758 | <0.01 | -3.021 | <0.01 |
| LPC20:5 | 0.633 (0.386) | 0.665 (0.770) | 0.376 (0.666) | 1.751 | 0.270 | 8.666 | <0.001 | 16.143 | <0.001 | 9.000 | <0.001 |
| LPC22:5 | -0.754 (0.529) | -0.871 (0.413) | -1.065 (0.505) | 2.953 | 0.106 | 26.821 | <0.001 | 37.640 | <0.001 | 18.466 | <0.001 |

Supplemental Table 2

Statistical test results revealing distinct concentrations of lipid species in NGT, IGT, and T2D individuals

Page 8

| Parameter  (µmol/l) | NGT  Mean (SD) | IGT  Mean (SD) | T2D  Mean (SD) | F-Statistic | p-value | IGT vs. NGT  (T-Statistic) | IGT vs. NGT  (p-value) | IGT vs. T2D  (T-Statistic) | IGT vs. T2D  (p-value) | NGT vs. T2D  (T-Statistic) | NGT vs. T2D  (p-value) |
| --- | --- | --- | --- | --- | --- | --- | --- | --- | --- | --- | --- |
| LPC22:6 | 1.253 (0.372) | 0.945 (0.454) | 0.853 (0.386) | 8.038 | 0.004 | 4.712 | <0.001 | 17.646 | <0.001 | 5.479 | <0.001 |
| DAG34:1 | 2.191 (0.366) | 2.439 (0.384) | 2.558 (0.335) | 7.832 | 0.005 | -10.362 | <0.001 | -0.639 | NS | -3.454 | 0.001 |
| DAG34:2 | 1.787 (0.375) | 2.012 (0.442) | 2.050 (0.391) | 3.804 | 0.058 | -4.976 | <0.001 | 4.748 | <0.001 | -0.270 | NS |
| DAG36:2 | 2.528 (0.318) | 2.703 (0.362) | 2.708 (0.337) | 2.846 | 0.116 | -14.754 | <0.001 | -3.299 | 0.001 | -5.371 | <0.001 |
| DAG36:3 | 2.378 (0.319) | 2.365 (0.321) | 2.400 (0.377) | 0.074 | 0.938 | -11.908 | <0.001 | 0.722 | NS | -3.788 | <0.001 |
| DAG36:4 | 1.463 (0.443) | 1.424 (0.272) | 1.509 (0.372) | 0.372 | 0.756 | 0.629 | NS | 12.424 | <0.001 | 2.674 | <0.05 |
| DAG38:5 | -0.273 (0.879) | 0.189 (0.661) | 0.133 (0.686) | 3.550 | 0.068 | 13.026 | <0.001 | 20.597 | <0.001 | 11.546 | <0.001 |
| PC30:0 | 0.148 (0.857) | 0.354 (0.628) | 0.506 (0.484) | 1.979 | 0.227 | 11.050 | <0.001 | 20.095 | <0.001 | 9.569 | <0.001 |
| PC32:0 | 1.976 (0.233) | 1.789 (0.296) | 1.790 (0.243) | 5.371 | 0.018 | -5.478 | <0.001 | 8.602 | <0.001 | -0.249 | NS |
| PC32:1 | 2.217 (0.374) | 2.493 (0.504) | 2.450 (0.399) | 3.779 | 0.059 | -10.163 | <0.001 | -0.396 | NS | -3.225 | <0.01 |
| PC32:2 | 0.865 (0.373) | 0.791 (0.307) | 0.832 (0.386) | 0.349 | 0.763 | 8.795 | <0.001 | 20.311 | <0.001 | 7.217 | <0.001 |
| PC33:1 | 0.681 (0.32) | 0.719 (0.272) | 0.717 (0.367) | 0.141 | 0.901 | 10.944 | <0.001 | 21.980 | <0.001 | 8.415 | <0.001 |
| PC34:1 | 5.000 (0.199) | 5.087 (0.210) | 5.033 (0.255) | 1.284 | 0.403 | -52.529 | <0.001 | -35.963 | <0.001 | -23.452 | <0.001 |

Supplemental Table 2

Statistical test results revealing distinct concentrations of lipid species in NGT, IGT, and T2D individuals

Page 9

| Parameter  (µmol/l) | NGT  Mean (SD) | IGT  Mean (SD) | T2D  Mean (SD) | F-Statistic | p-value | IGT vs. NGT  (T-Statistic) | IGT vs. NGT  (p-value) | IGT vs. T2D  (T-Statistic) | IGT vs. T2D  (p-value) | NGT vs. T2D  (T-Statistic) | NGT vs. T2D  (p-value) |
| --- | --- | --- | --- | --- | --- | --- | --- | --- | --- | --- | --- |
| PC34:2 | 5.886 (0.228) | 5.679 (0.271) | 5.678 (0.282) | 6.687 | 0.009 | -60.582 | <0.001 | -42.878 | <0.001 | -28.943 | <0.001 |
| PC34:3 | 2.441 (0.321) | 2.356 (0.264) | 2.348 (0.457) | 0.682 | 0.626 | -12.519 | <0.001 | 1.060 | NS | -3.831 | <0.001 |
| PC34:4 | -0.024 (0.500) | 0.116 (0.340) | 0.087 (0.346) | 1.060 | 0.473 | 17.594 | <0.001 | 29.136 | <0.001 | 12.824 | <0.001 |
| PC35:1 | 0.416 (0.325) | 0.493 (0.247) | 0.424 (0.361) | 0.584 | 0.665 | 14.406 | <0.001 | 25.534 | <0.001 | 10.417 | <0.001 |
| PC35:2 | 1.970 (0.324) | 1.650 (0.425) | 1.619 (0.350) | 8.561 | 0.003 | -3.988 | <0.001 | 9.534 | <0.001 | 0.308 | NS |
| PC36:2 | 5.248 (0.249) | 4.985 (0.234) | 4.963 (0.240) | 13.435 | 0.000 | -50.985 | <0.001 | -34.643 | <0.001 | -23.942 | <0.001 |
| PC36:3 | 4.441 (0.256) | 4.332 (0.245) | 4.320 (0.277) | 2.079 | 0.210 | -41.364 | <0.001 | -25.334 | <0.001 | -18.598 | <0.001 |
| PC36:4 | 5.124 (0.236) | 5.092 (0.311) | 5.022 (0.216) | 1.134 | 0.450 | -50.844 | <0.001 | -34.716 | <0.001 | -23.897 | <0.001 |
| PC36:5 | 3.300 (0.412) | 3.407 (0.586) | 3.257 (0.554) | 0.653 | 0.640 | -21.044 | <0.001 | -8.966 | <0.001 | -9.774 | <0.001 |
| PC37:2 | -0.525 (0.341) | -0.960 (0.385) | -0.898 (0.367) | 13.324 | 0.000 | 27.371 | <0.001 | 40.697 | <0.001 | 18.054 | <0.001 |
| PC37:4 | 1.052 (0.371) | 0.917 (0.529) | 0.828 (0.347) | 2.026 | 0.219 | 6.007 | <0.001 | 17.392 | <0.001 | 6.470 | <0.001 |
| PC38:3 | 2.367 (0.475) | 2.611 (0.414) | 2.701 (0.390) | 4.824 | 0.027 | -11.760 | <0.001 | -2.486 | <0.05 | -4.478 | <0.001 |
| PC38:4 | 4.416 (0.326) | 4.377 (0.354) | 4.289 (0.255) | 1.156 | 0.446 | -38.606 | <0.001 | -24.253 | <0.001 | -18.236 | <0.001 |

Supplemental Table 2

Statistical test results revealing distinct concentrations of lipid species in NGT, IGT, and T2D individuals

Page 10

| Parameter  (µmol/l) | NGT  Mean (SD) | IGT  Mean (SD) | T2D  Mean (SD) | F-Statistic | p-value | IGT vs. NGT  (T-Statistic) | IGT vs. NGT  (p-value) | IGT vs. T2D  (T-Statistic) | IGT vs. T2D  (p-value) | NGT vs. T2D  (T-Statistic) | NGT vs. T2D  (p-value) |
| --- | --- | --- | --- | --- | --- | --- | --- | --- | --- | --- | --- |
| PC38:5 | 3.510 (0.279) | 3.631 (0.276) | 3.517 (0.296) | 1.808 | 0.260 | -29.006 | <0.001 | -14.933 | <0.001 | -12.104 | <0.001 |
| PC38:6 | 4.419 (0.299) | 4.296 (0.351) | 4.239 (0.312) | 2.427 | 0.160 | -38.405 | <0.001 | -23.012 | <0.001 | -18.023 | <0.001 |
| PC38:7 | 0.269 (0.308) | 0.232 (0.515) | 0.177 (0.397) | 0.346 | 0.763 | 15.266 | <0.001 | 24.679 | <0.001 | 11.750 | <0.001 |
| PC40:4 | 0.184 (0.412) | 0.262 (0.387) | 0.259 (0.336) | 0.420 | 0.733 | 15.928 | <0.001 | 26.602 | <0.001 | 11.727 | <0.001 |
| PC40:5 | 0.659 (0.772) | 1.074 (0.547) | 0.967 (0.678) | 3.296 | 0.081 | 5.892 | <0.001 | 13.574 | <0.001 | 6.544 | <0.001 |
| PC40:6 | 3.172 (0.343) | 3.065 (0.382) | 3.110 (0.341) | 0.729 | 0.605 | -21.102 | <0.001 | -7.898 | <0.001 | -9.259 | <0.001 |
| PC40:7 | 1.407 (0.303) | 1.284 (0.336) | 1.207 (0.287) | 3.079 | 0.097 | 1.965 | NS | 15.163 | <0.001 | 3.941 | <0.001 |
| PC40:8 | -0.423 (0.705) | -0.038 (0.793) | 0.024 (0.669) | 3.398 | 0.076 | 15.141 | <0.001 | 20.789 | <0.001 | 12.944 | <0.001 |
| PC O-32:0 | -0.334 (0.374) | -0.551 (0.440) | -0.709 (0.356) | 6.646 | 0.009 | 23.772 | <0.001 | 35.708 | <0.001 | 16.631 | <0.001 |
| PC O-32:1 | 0.280 (0.267) | 0.136 (0.295) | 0.025 (0.265) | 6.172 | 0.012 | 17.827 | <0.001 | 30.812 | <0.001 | 12.377 | <0.001 |
| PC O-34:1 | 0.651 (0.275) | 0.384 (0.303) | 0.300 (0.266) | 12.716 | 0.000 | 13.177 | <0.001 | 27.342 | <0.001 | 9.888 | <0.001 |
| PC O-34:2 | 1.397 (0.351) | 1.188 (0.422) | 1.032 (0.315) | 7.179 | 0.007 | 2.486 | <0.05 | 15.789 | <0.001 | 4.433 | <0.001 |
| PC O-34:3 | 1.668 (0.327) | 1.328 (0.418) | 1.184 (0.336) | 13.895 | 0.000 | -0.106 | NS | 13.993 | <0.001 | 2.841 | <0.01 |

Supplemental Table 2

Statistical test results revealing distinct concentrations of lipid species in NGT, IGT, and T2D individuals

Page 11

| Parameter  (µmol/l) | NGT  Mean (SD) | IGT  Mean (SD) | T2D  Mean (SD) | F-Statistic | p-value | IGT vs. NGT  (T-Statistic) | IGT vs. NGT  (p-value) | IGT vs. T2D  (T-Statistic) | IGT vs. T2D  (p-value) | NGT vs. T2D  (T-Statistic) | NGT vs. T2D  (p-value) |
| --- | --- | --- | --- | --- | --- | --- | --- | --- | --- | --- | --- |
| PC O-36:2 | 0.241 (0.342) | -0.090 (0.392) | -0.228 (0.280) | 14.524 | 0.000 | 17.708 | <0.001 | 31.956 | <0.001 | 12.939 | <0.001 |
| PC O-36:3 | 1.077 (0.353) | 0.845 (0.435) | 0.700  (0.343) | 7.258 | 0.006 | 6.573 | <0.001 | 19.506 | <0.001 | 6.720 | <0.001 |
| PC O-36:4 | 2.212 (0.324) | 2.127 (0.424) | 1.972 (0.279) | 3.330 | 0.079 | -8.692 | <0.001 | 4.595 | <0.001 | -1.785 | NS |
| PC O-36:5 | 2.146 (0.287) | 2.067 (0.346) | 1.871 (0.263) | 6.006 | 0.013 | -8.326 | <0.001 | 5.785 | <0.001 | -1.206 | NS |
| PC O-36:6 | -0.601 (0.692) | -0.627 (0.663) | -0.924 (0.633) | 1.974 | 0.227 | 20.206 | <0.001 | 29.099 | <0.001 | 16.394 | <0.001 |
| PC O-38:4 | 1.031 (0.420) | 0.825 (0.452) | 0.791 (0.270) | 3.279 | 0.081 | 6.756 | <0.001 | 19.565 | <0.001 | 6.661 | <0.001 |
| PC O-38:5 | 2.333 (0.306) | 2.212 (0.394) | 2.040 (0.264) | 5.565 | 0.016 | -10.222 | <0.001 | 3.722 | <0.001 | -2.500 | <0.05 |
| PC O-38:6 | 1.557 (0.289) | 1.437 (0.324) | 1.329 (0.282) | 4.133 | 0.046 | -0.074 | NS | 13.465 | <0.001 | 2.942 | <0.01 |
| PC O-38:7 | 0.353 (0.445) | 0.225 (0.449) | 0.089 (0.354) | 2.788 | 0.120 | 14.370 | <0.001 | 26.463 | <0.001 | 11.379 | <0.001 |
| PC O-40:7 | 0.620 (0.364) | 0.393 (0.423) | 0.317 (0.330) | 5.233 | 0.020 | 12.245 | <0.001 | 24.888 | <0.001 | 9.786 | <0.001 |
| PI34:1 | 0.376 (0.392) | 0.608 (0.545) | 0.699 (0.476) | 3.703 | 0.063 | 11.563 | <0.001 | 18.848 | <0.001 | 9.341 | <0.001 |
| PI34:2 | 0.354 (0.391) | 0.274 (0.556) | 0.481 (0.390) | 1.449 | 0.351 | 13.684 | <0.001 | 22.200 | <0.001 | 10.315 | <0.001 |
| PI36:1 | 0.736 (0.354) | 0.746 (0.281) | 0.697 (0.304) | 0.182 | 0.879 | 10.179 | <0.001 | 22.380 | <0.001 | 8.272 | <0.001 |

Supplemental Table 2

Statistical test results revealing distinct concentrations of lipid species in NGT, IGT, and T2D individuals

Page 12

| Parameter  (µmol/l) | NGT  Mean (SD) | IGT  Mean (SD) | T2D  Mean (SD) | F-Statistic | p-value | IGT vs. NGT  (T-Statistic) | IGT vs. NGT  (p-value) | IGT vs. T2D  (T-Statistic) | IGT vs. T2D  (p-value) | NGT vs. T2D  (T-Statistic) | NGT vs. T2D  (p-value) |
| --- | --- | --- | --- | --- | --- | --- | --- | --- | --- | --- | --- |
| PI36:2 | 1.746 (0.332) | 1.611 (0.379) | 1.652 (0.313) | 1.305 | 0.397 | -2.437 | <0.05 | 10.016 | <0.001 | 1.136 | NS |
| PI36:3 | -0.033 (0.384) | -0.081 (0.402) | -0.053 (0.261) | 0.143 | 0.901 | 19.587 | <0.001 | 31.139 | <0.001 | 13.762 | <0.001 |
| PI36:4 | 0.741 (0.221) | 0.894 (0.309) | 0.892 (0.250) | 3.481 | 0.071 | 9.473 | <0.001 | 20.297 | <0.001 | 7.818 | <0.001 |
| PI38:4 | 3.230 (0.220) | 3.239 (0.248) | 3.222 (0.248) | 0.038 | 0.963 | -25.318 | <0.001 | -10.730 | <0.001 | -10.135 | <0.001 |
| PI38:5 | 0.556 (0.193) | 0.522 (0.281) | 0.380 (0.180) | 4.684 | 0.030 | 13.782 | <0.001 | 26.693 | <0.001 | 10.319 | <0.001 |
| PI38:6 | -0.774 (0.402) | -0.704 (0.506) | -0.624 (0.408) | 0.815 | 0.576 | 26.532 | <0.001 | 34.385 | <0.001 | 18.148 | <0.001 |
| PI40:5 | -0.805 (0.355) | -0.585 (0.337) | -0.595 (0.421) | 3.555 | 0.068 | 28.470 | <0.001 | 36.629 | <0.001 | 18.260 | <0.001 |
| PI40:6 | 0.222 (0.407) | 0.188 (0.395) | 0.295 (0.424) | 0.494 | 0.701 | 16.161 | <0.001 | 25.766 | <0.001 | 11.337 | <0.001 |
| PE34:1 | -1.246 (0.426) | -0.871 (0.719) | -0.741 (0.535) | 6.278 | 0.011 | 25.927 | <0.001 | 30.152 | <0.001 | 19.428 | <0.001 |
| PE34:2 | 0.090  (0.280) | 0.141 (0.446) | 0.219 (0.406) | 0.812 | 0.576 | 17.777 | <0.001 | 25.969 | <0.001 | 12.376 | <0.001 |
| PE36:2 | 1.099 (0.257) | 1.137 (0.389) | 1.196 (0.370) | 0.584 | 0.665 | 5.033 | <0.001 | 15.351 | <0.001 | 5.249 | <0.001 |
| PE36:3 | -0.532 (0.359) | -0.587 (0.591) | -0.543 (0.679) | 0.088 | 0.934 | 23.668 | <0.001 | 28.334 | <0.001 | 16.246 | <0.001 |
| PE36:4 | 0.432 (0.299) | 0.613 (0.420) | 0.664 (0.333) | 3.567 | 0.068 | 12.503 | <0.001 | 21.622 | <0.001 | 9.620 | <0.001 |

Supplemental Table 2

Statistical test results revealing distinct concentrations of lipid species in NGT, IGT, and T2D individuals

Page 13

| Parameter  (µmol/l) | NGT  Mean (SD) | IGT  Mean (SD) | T2D  Mean (SD) | F-Statistic | p-value | IGT vs. NGT  (T-Statistic) | IGT vs. NGT  (p-value) | IGT vs. T2D  (T-Statistic) | IGT vs. T2D  (p-value) | NGT vs. T2D  (T-Statistic) | NGT vs. T2D  (p-value) |
| --- | --- | --- | --- | --- | --- | --- | --- | --- | --- | --- | --- |
| PE38:4 | 1.329 (0.304) | 1.547 (0.304) | 1.606 (0.305) | 6.971 | 0.007 | 0.750 | NS | 11.162 | <0.001 | 2.990 | <0.01 |
| PE38:5 | 0.185 (0.281) | 0.475 (0.436) | 0.389 (0.332) | 5.680 | 0.015 | 14.724 | <0.001 | 23.746 | <0.001 | 11.544 | <0.001 |
| PE38:6 | 0.765 (0.548) | 1.055 (0.548) | 1.192 (0.381) | 5.464 | 0.017 | 6.234 | <0.001 | 14.362 | <0.001 | 6.160 | <0.001 |
| PE40:6 | 0.372 (0.517) | 0.687 (0.481) | 0.865 (0.391) | 8.242 | 0.004 | 10.736 | <0.001 | 18.782 | <0.001 | 8.621 | <0.001 |
| PE40:7 | -0.720 (0.309) | -0.627 (0.375) | -0.644 (0.238) | 0.768 | 0.590 | 28.643 | <0.001 | 39.066 | <0.001 | 18.707 | <0.001 |
| PE O-34:3 | 0.053 (0.375) | -0.147 (0.448) | -0.221 (0.278) | 4.154 | 0.045 | 18.878 | <0.001 | 31.402 | <0.001 | 13.820 | <0.001 |
| PE O-36:3 | 0.486 (0.344) | 0.269 (0.439) | 0.204 (0.304) | 4.791 | 0.028 | 13.884 | <0.001 | 26.313 | <0.001 | 10.764 | <0.001 |
| PE O-36:4 | 0.380 (0.359) | 0.220 (0.422) | 0.159 (0.262) | 3.003 | 0.103 | 14.980 | <0.001 | 27.516 | <0.001 | 11.396 | <0.001 |
| PE O-36:5 | 1.468 (0.363) | 1.362 (0.428) | 1.265 (0.317) | 2.102 | 0.207 | 0.960 | NS | 13.405 | <0.001 | 3.455 | 0.001 |
| PE O-36:6 | -0.480 (0.716) | -0.482 (0.764) | -0.639 (0.632) | 0.441 | 0.726 | 17.872 | <0.001 | 26.045 | <0.001 | 15.189 | <0.001 |
| PE O-38:4 | -0.952 (0.584) | -1.085 (0.526) | -1.082 (0.358) | 0.700 | 0.619 | 26.974 | <0.001 | 39.274 | <0.001 | 19.567 | <0.001 |
| PE O-38:5 | 1.703 (0.359) | 1.568 (0.495) | 1.471 (0.299) | 2.477 | 0.154 | -1.750 | NS | 10.550 | <0.001 | 1.863 | NS |
| PE O-38:6 | 1.764 (0.343) | 1.701 (0.384) | 1.557 (0.276) | 2.671 | 0.133 | -3.123 | <0.01 | 9.941 | <0.001 | 1.359 | NS |

Supplemental Table 2

Statistical test results revealing distinct concentrations of lipid species in NGT, IGT, and T2D individuals

Page 14

| Parameter  (µmol/l) | NGT  Mean (SD) | IGT  Mean (SD) | T2D  Mean (SD) | F-Statistic | p-value | IGT vs. NGT  (T-Statistic) | IGT vs. NGT  (p-value) | IGT vs. T2D  (T-Statistic) | IGT vs. T2D  (p-value) | NGT vs. T2D  (T-Statistic) | NGT vs. T2D  (p-value) |
| --- | --- | --- | --- | --- | --- | --- | --- | --- | --- | --- | --- |
| PE O-38:7 | 1.332 (0.393) | 1.258 (0.357) | 1.201 (0.298) | 0.989 | 0.500 | 2.525 | <0.05 | 15.170 | <0.001 | 4.208 | <0.001 |
| PE O-40:7 | 0.860 (0.399) | 0.658 (0.397) | 0.602 (0.303) | 3.991 | 0.050 | 9.106 | <0.001 | 22.171 | <0.001 | 7.934 | <0.001 |
| PE O-40:8 | 0.503 (0.459) | 0.331 (0.400) | 0.245 (0.366) | 2.988 | 0.103 | 12.991 | <0.001 | 25.664 | <0.001 | 10.267 | <0.001 |
| SM32:1:1 | 2.617 (0.236) | 2.393 (0.293) | 2.352 (0.256) | 9.026 | 0.003 | -14.106 | <0.001 | 0.843 | NS | -4.706 | <0.001 |
| SM32:2:1 | -0.377 (0.328) | -0.776 (0.634) | -0.823 (0.501) | 7.350 | 0.006 | 22.607 | <0.001 | 32.032 | <0.001 | 16.807 | <0.001 |
| SM33:1:1 | 1.926 (0.265) | 1.661 (0.416) | 1.607 (0.345) | 7.395 | 0.006 | -3.909 | <0.001 | 9.590 | <0.001 | 0.532 | NS |
| SM34:1:1 | 4.845 (0.228) | 4.606 (0.327) | 4.486 (0.279) | 12.586 | 0.000 | -44.341 | <0.001 | -27.117 | <0.001 | -20.612 | <0.001 |
| SM34:2:1 | 2.985 (0.195) | 2.739 (0.294) | 2.658 (0.254) | 13.949 | 0.000 | -19.247 | <0.001 | -3.493 | 0.001 | -7.219 | <0.001 |
| SM35:1:1 | 1.172 (0.271) | 0.918 (0.443) | 0.862 (0.398) | 5.906 | 0.013 | 5.664 | <0.001 | 17.833 | <0.001 | 5.902 | <0.001 |
| SM36:1:1 | 3.119 (0.236) | 3.021 (0.330) | 2.855 (0.314) | 5.743 | 0.015 | -21.928 | <0.001 | -6.415 | <0.001 | -8.345 | <0.001 |
| SM36:2:1 | 2.512 (0.232) | 2.379 (0.332) | 2.187 (0.307) | 8.840 | 0.003 | -13.217 | <0.001 | 1.810 | NS | -3.710 | 0.001 |
| SM36:3:1 | -0.652 (0.598) | -0.779 (0.615) | -1.230 (0.528) | 7.389 | 0.006 | 22.595 | <0.001 | 32.471 | <0.001 | 17.790 | <0.001 |
| SM37:1:1 | 0.418 (0.394) | 0.240 (0.515) | 0.187 (0.447) | 2.152 | 0.199 | 13.693 | <0.001 | 24.125 | <0.001 | 10.807 | <0.001 |

Supplemental Table 2

Statistical test results revealing distinct concentrations of lipid species in NGT, IGT, and T2D individuals

Page 15

| Parameter  (µmol/l) | NGT  Mean (SD) | IGT  Mean (SD) | T2D  Mean (SD) | F-Statistic | p-value | IGT vs. NGT  (T-Statistic) | IGT vs. NGT  (p-value) | IGT vs. T2D  (T-Statistic) | IGT vs. T2D  (p-value) | NGT vs. T2D  (T-Statistic) | NGT vs. T2D  (p-value) |
| --- | --- | --- | --- | --- | --- | --- | --- | --- | --- | --- | --- |
| SM38:1:1 | 2.772 (0.200) | 2.577 (0.315) | 2.498 (0.285) | 8.180 | 0.004 | -16.569 | <0.001 | -1.338 | NS | -5.818 | <0.001 |
| SM38:2:1 | 1.866 (0.233) | 1.662 (0.328) | 1.543 (0.303) | 9.427 | 0.002 | -3.761 | <0.001 | 10.532 | <0.001 | 0.983 | NS |
| SM39:1:1 | 1.666 (0.263) | 1.426 (0.440) | 1.428 (0.330) | 4.781 | 0.028 | -0.709 | NS | 12.009 | <0.001 | 2.172 | <0.05 |
| SM39:2:1 | -0.151 (0.353) | -0.352 (0.385) | -0.515 (0.513) | 5.617 | 0.016 | 22.265 | <0.001 | 32.142 | <0.001 | 15.014 | <0.001 |
| SM40:1:1 | 3.263 (0.234) | 3.060 (0.291) | 3.027 (0.294) | 6.733 | 0.008 | -23.346 | <0.001 | -7.916 | <0.001 | -9.523 | <0.001 |
| SM40:2:1 | 3.236 (0.169) | 2.979 (0.334) | 2.899 (0.265) | 13.406 | 0.000 | -22.388 | <0.001 | -6.502 | <0.001 | -9.038 | <0.001 |
| SM40:3:1 | 0.629 (0.301) | 0.254 (0.600) | 0.101 (0.770) | 6.770 | 0.008 | 11.841 | <0.001 | 20.177 | <0.001 | 9.512 | <0.001 |
| SM41:1:1 | 2.439 (0.252) | 2.219 (0.339) | 2.170 (0.305) | 6.959 | 0.007 | -11.368 | <0.001 | 3.055 | <0.01 | -3.361 | <0.01 |
| SM41:2:1 | 2.296 (0.257) | 2.046 (0.389) | 1.936 (0.386) | 8.512 | 0.003 | -8.943 | <0.001 | 5.260 | <0.001 | -1.994 | NS |
| SM41:3:1 | 0.144 (0.274) | -0.077 (0.335) | -0.196 (0.339) | 8.833 | 0.003 | 19.628 | <0.001 | 32.061 | <0.001 | 13.430 | <0.001 |
| SM42:1:1 | 2.042 (0.587) | 1.87 (0.531) | 1.911 (0.658) | 0.751 | 0.596 | -4.967 | <0.001 | 5.397 | <0.001 | -0.803 | NS |
| SM42:2:1 | 4.181 (0.213) | 4.018 (0.300) | 3.890 (0.305) | 8.270 | 0.004 | -36.790 | <0.001 | -19.597 | <0.001 | -16.046 | <0.001 |
| SM42:3:1 | 3.441 (0.211) | 3.207 (0.341) | 3.083 (0.298) | 11.822 | 0.001 | -25.113 | <0.001 | -9.024 | <0.001 | -10.379 | <0.001 |

Supplemental Table 2

Statistical test results revealing distinct concentrations of lipid species in NGT, IGT, and T2D individuals

Page 16

| Parameter  (µmol/l) | NGT  Mean (SD) | IGT  Mean (SD) | T2D  Mean (SD) | F-Statistic | p-value | IGT vs. NGT  (T-Statistic) | IGT vs. NGT  (p-value) | IGT vs. T2D  (T-Statistic) | IGT vs. T2D  (p-value) | NGT vs. T2D  (T-Statistic) | NGT vs. T2D  (p-value) |
| --- | --- | --- | --- | --- | --- | --- | --- | --- | --- | --- | --- |
| SM42:4:1 | 0.944 (0.327) | 0.733 (0.331) | 0.591 (0.289) | 9.107 | 0.003 | 8.618 | <0.001 | 22.488 | <0.001 | 7.683 | <0.001 |
| SM43:2:1 | 0.0002 (0.998) | 0.0068  (0.804) | -0.0089  (1.243) | 0.002 | 0.998 | -0.030 | NS | 0.055 | NS | 0.030 | NS |
|  |  |  |  |  |  |  |  |  |  |  |  |
|  |  |  |  |  |  |  |  |  |  |  |  |
|  |  |  |  |  |  |  |  |  |  |  |  |
|  |  |  |  |  |  |  |  |  |  |  |  |
|  |  |  |  |  |  |  |  |  |  |  |  |
|  |  |  |  |  |  |  |  |  |  |  |  |
|  |  |  |  |  |  |  |  |  |  |  |  |
|  |  |  |  |  |  |  |  |  |  |  |  |
|  |  |  |  |  |  |  |  |  |  |  |  |
