# Supplementary material for: Detection of Independent Associations of Plasma Lipidomic Parameters with Insulin Sensitivity Indices Using Data Mining Methodology
Source: PLoS One. 2016 Oct 13;11(10):e0164173. doi: 10.1371/journal.pone.0164173 (PMC5063331; doi:10.1371/journal.pone.0164173)
Supplement: S3 Table — Overall tests for differential concentrations was conducted by ANOVA, pairwise comparisons by t-tests. (DOCX) [file pone.0164173.s005.docx]

Supplemental Table 3

Page 1

Correlations of lipid species with different insulin sensitivity indices

| Lipid species | HOMA-IR | GSI | ISI | DI |
| --- | --- | --- | --- | --- |
|  | Spearman’s nonparametric rank correlation coefficient  (significance) | | | |
| **Triacylglycerols (TAG)**  TAG 44:0  TAG 46:0  TAG 46:1  TAG 46:2  TAG 47:1  TAG 47:2  TAG 48:1  TAG 48:2  TAG 48:3  TAG 49:1  TAG 49:2  TAG 49:3  TAG 50:1  TAG 50:2  TAG 50:3  TAG 50:4  TAG 50:5  TAG 51:1  TAG 51:2  TAG 51:3  TAG 51:4 | 0.366  (0.001)  0.463  (<0.001)  0.444  (<0.001)  0.413  (<0.001)  0.413  (<0.001)  0.332  (0.004)  0.459  (<0.001)  0.410  (<0.001)  0.440  (<0.001)  0.457  (<0.001)  0.390  (<0.001)  0.371  (0.001)  0.494  (<0.001)  0.410  (<0.001)  0.365  (0.001)  0.346  (0.003)  0.390  (<0.001)  0.489  (<0.001)  0.387  (<0.001)  0.279  (<0.019)  0.183  (0.131) | -0.229  (0.074)  -0.291  (0.024)  -0.257  (0.046)  -0.210  (0.099)  -0.249  (0.053)  -0.186  (0.147)  -0.307  (0.018)  -0.262  (0.042)  -0.253  (0.050)  -0.348  (0.009)  -0.319  (0.015)  -0.288  (0.026)  -0.360  (0.008)  -0.334  (0.012)  -0.326  (0.012)  -0.233  (0.068)  -0.234  (0.068)  -0.337  (0.011)  -0.315  (0.015)  -0.271  (0.035)  -0.206  (0.107) | -0.395  (<0.001)  -0.483  (<0.001)  -0.452  (<0.001)  -0.400  (<0.001)  -0.394  (<0.001)  -0.343  (0.002)  -0.483  (<0.001)  -0.437  (<0.001)  -0.407  (<0.001)  -0.426  (<0.001)  -0.406  (<0.001)  -0.412  (<0.001)  -0.508  (<0.001)  -0.454  (<0.001)  -0.440  (<0.001)  -0.388  (<0.001)  -0.399  (<0.001)  -0.415  (<0.001)  -0.412  (<0.001)  -0.289  (0.011)  -0.220  (0.060) | -0.226  (0.072)  -0.291  (0.022)  -0.274  (0.031)  -0.232  (0.065)  -0.219  (0.080)  -0.194  (0.120)  -0.291  (0.022)  -0.265  (0.037)  -0.217  (0.083)  -0.210  (0.093)  -0.226  (0.072)  -0.238  (0.059)  -0.287  (0.024)  -0.301  (0.019)  -0.297  (0.020)  -0.247  (0.051)  -0.224  (0.074)  -0.152  (0.237)  -0.207  (0.098)  -0.155  (0.230)  -0.051  (0.704) |

Supplemental Table 3

Page 2

Correlations of lipid species with different insulin sensitivity indices

| Lipid species | HOMA-IR | GSI | ISI | DI |
| --- | --- | --- | --- | --- |
|  | Spearman’s nonparametric rank correlation coefficient  (significance) | | | |
| **Triacylglycerols (TAG)**  TAG 51:5  TAG 52:2  TAG 52:3  TAG 52:4  TAG 52:5  TAG 52:6  TAG 52:7  TAG 53:2  TAG 53:3  TAG 53:4  TAG 53:5  TAG 53:6  TAG 53:7  TAG 54:1  TAG 54:2  TAG 54:3  TAG 54:4  TAG 54:5  TAG 54:6  TAG 54:7  TAG 54:8 | 0.334  (0.004)  0.419  (<0.001)  0.163  (0.183)  0.089  (0.468)  0.156  (0.206)  0.276  (0.020)  0.289  (0.015)  0.312  (0.007)  0.240  (0.044)  0.132  (0.281)  0.238  (0.046)  0.317  (0.006)  0.147  (0.234)  0.260  (0.030)  0.504  (<0.001)  0.204  (0.090)  0.052  (0.680)  0.105  (0.400)  0.177  (0.144)  0.183  (0.131)  0.189  (0.120) | -0.230  (0.072)  -0.315  (0.015)  -0.198  (0.121)  -0.177  (0.173)  -0.239  (0.063)  -0.156  (0.241)  -0.106  (0.431)  -0.275  (0.033)  -0.277  (0.033)  -0.216  (0.089)  -0.249  (0.053)  -0.272  (0.034)  -0.226  (0.075)  -0.223  (0.079)  -0.318  (0.015)  -0.128  (0.340)  -0.102  (0.440)  -0.153  (0.249)  -0.191  (0.134)  -0.096  (0.462)  -0.106  (0.431) | -0.437  (<0.001)  -0.410  (<0.001)  -0.193  (0.101)  -0.134  (0.259)  -0.199  (0.090)  -0.295  (0.010)  -0.301  (0.008)  -0.278  (0.015)  -0.183  (0.119)  -0.152  (0.195)  -0.224  (0.055)  -0.413  (<0.001)  -0.252  (0.029)  -0.169  (0.149)  -0.379  (<0.001)  -0.136  (0.253)  0.013  (0.912)  -0.094  (0.436)  -0.215  (0.067)  -0.180  (0.126)  -0.155  (0.188) | -0.334  (0.011)  -0.259  (0.042)  -0.114  (0.395)  -0.067  (0.639)  -0.140  (0.277)  -0.252  (0.047)  -0.243  (0.054)  -0.110  (0.406)  -0.040  (0.777)  -0.037  (0.787)  -0.064  (0.639)  -0.343  (0.010)  -0.219  (0.080)  -0.057  (0.675)  -0.139  (0.280)  -0.074  (0.592)  0.061  (0.651)  -0.028  (0.832)  -0.179  (0.158)  -0.170  (0.181)  -0.144  (0.262) |

Supplemental Table 3

Page 3

Correlations of lipid species with different insulin sensitivity indices

| Lipid species | HOMA-IR | GSI | ISI | DI |
| --- | --- | --- | --- | --- |
|  | Spearman’s nonparametric rank correlation coefficient  (significance) | | | |
| **Triacylglycerols (TAG)**  TAG 55:6  TAG 55:7  TAG 56:3  TAG 56:4  TAG 56:5  TAG 56:6  TAG 56:7  TAG 56:8  TAG 56:9  TAG 58:6  TAG 58:7  TAG 58:8  TAG 58:9  TAG 58:10  **Diacylglycerols (DAG)**  DAG 34:1  DAG 34:2  DAG 36:2  DAG 36:3  DAG 36:4  DAG 38:5 | 0.277  (0.020)  0.275  (0.021)  0.226  (0.058)  0.184  (0.131)  0.180  (0.139)  0.080  (0.515)  0.250  (0.037)  0.142  (0.249)  0.183  (0.131)  0.333  (0.004)  0.343  (0.003)  0.129  (0.291)  0.116  (0.346)  0.121  (0.324)  0.397  (<0.001)  0.239  (0.045)  0.233  (0.051)  0.049  (0.694)  0.073  (0.551)  0.259  (0.031) | -0.200  (0.120)  -0.210  (0.099)  -0.115  (0.394)  -0.138  (0.301)  -0.213  (0.094)  -0.123  (0.357)  -0.234  (0.068)  -0.096  (0.462)  -0.134  (0.317)  -0.315  (0.015)  -0.329  (0.012)  -0.096  (0.462)  -0.071  (0.582)  -0.054  (0.684)  -0.319  (0.015)  -0.249  (0.053)  -0.243  (0.058)  -0.154  (0.243)  -0.164  (0.215)  -0.246  (0.055) | -0.336  (0.003)  -0.286  (0.012)  -0.206  (0.080)  -0.185  (0.118)  -0.180  (0.126)  -0.179  (0.127)  -0.239  (0.039)  -0.092  (0.448)  -0.140  (0.235)  -0.467  (<0.001)  -0.393  (<0.001)  -0.107  (0.371)  -0.097  (0.424)  -0.085  (0.482)  -0.420  (<0.001)  -0.310  (0.006)  -0.262  (0.023)  -0.044  (0.719)  -0.079  (0.511)  0.345  (0.002) | -0.232  (0.065)  -0.241  (0.056)  -0.198  (0.113)  -0.164  (0.199)  -0.125  (0.337)  -0.264  (0.038)  -0.206  (0.098)  -0.064  (0.639)  -0.108  (0.416)  -0.403  (0.003)  -0.327  (0.012)  -0.110  (0.408)  -0.064  (0.639)  -0.090  (0.513)  -0.324  (0.012)  -0.288  (0.023)  -0.282  (0.025)  -0.038  (0.786)  -0.064  (0.639)  -0.256  (0.044) |

Supplemental Table 3

Page 4

Correlations of lipid species with different insulin sensitivity indices

| Lipid species | HOMA-IR | GSI | ISI | DI |
| --- | --- | --- | --- | --- |
|  | Spearman’s nonparametric rank correlation coefficient  (Significance) | | | |
| **Cholesterylester (CE)**  CE 14:0  CE 15:0  CE 16:0  CE 16:1  CE 17:1  CE 18:1  CE 18:2  CE 18:3  CE 19:2  CE 20:3  CE 20:4  CE 20:5  CE 22:6  **Ceramides (Cer)**  Cer 40:1:2  Cer 41:1:2  Cer 42:1:2  Cer 42:2:2  **Phosphatidylcholines (PC)**  PC 30:0  PC 32:0  PC 32:1 | 0.136  (0.270)  -0.099  (0.429)  -0.169  (0.166)  0.141  (0.252)  -0.032  (0.794)  -0.010  (0.934)  -0.090  (0.488)  -0.037  (0.771)  -0.425  (<0.001)  0.152  (0.220)  -0.210  (0.083)  -0.020  (0.864)  -0.245  (0.040)  0.086  (0.482)  0.088  (0.470)  -0.177  (0.144)  -0.151  (0.223)  0.095  (0.439)  -0.434  (<0.001)  0.097  (0.431) | -0.148  (0.264)  0.052  (0.691)  -0.221  (0.083)  -0.130  (0.334)  0.069  (0.597)  -0.057  (0.666)  -0.144  (0.241)  0.077  (0.594)  0.342  (0.010)  -0.220  (0.083)  0.061  (0.636)  0.123  (0.357)  0.227  (0.075)  -0.115  (0.394)  -0.149  (0.263)  0.101  (0.442)  0.111  (0.417)  -0.043  (0.747)  0.368  (0.007)  -0.037  (0.783) | -0.127  (0.287)  0.161  (0.169)  0.234  (0.044)  -0.245  (0.034)  0.057  (0.640)  -0.036  (0.767)  0.148  (0.239)  0.012  (0.919)  0.556  (<0.001)  -0.094  (0.436)  0.153  (0.192)  0.072  (0.549)  0.308  (0.007)  -0.128  (0.282)  -0.168  (0.152)  0.119  (0.321)  0.055  (0.650)  -0.146  (0.217)  0.442  (<0.001)  -0.193  (0.101) | -0.132  (0.310)  0.177  (0.163)  0.054  (0.691)  -0.289  (0.023)  0.028  (0.832)  0.074  (0.592)  -0.047  (0.976)  -0.013  (0.927)  0.333  (0.011)  0.035  (0.800)  0.063  (0.640)  0.022  (0.871)  0.110  (0.406)  -0.093  (0.499)  -0.030  (0.823)  0.054  (0.691)  -0.112  (0.399)  -0.085  (0.536)  -0.260  (0.042)  -0.245  (0.053) |

Supplemental Table 3

Page 5

Correlations of lipid species with different insulin sensitivity indices

| Lipid species | HOMA-IR | GSI | ISI | DI |
| --- | --- | --- | --- | --- |
|  | Spearman’s nonparametric rank correlation coefficient  (Significance) | | | |
| **Phosphatidylcholines (PC)**  PC 32:2  PC 33:1  PC 34:1  PC 34:2  PC 34:3  PC 34:4  PC 35:1  PC 35:2  PC 36:2  PC 36:3  PC 36:4  PC 36:5  PC 37:2  PC 37:4  PC 38:3  PC 38:4  PC 38:5  PC 38:6  PC 38:7  PC 40:4  PC 40:5 | 0.056  (0.664)  0.048  (0.694)  -0.080  (0.515)  -0.190  (0.118)  -0.176  (0.146)  0.140  (0.254)  -0.050  (0.691)  -0.366  (0.001)  -0.264  (0.027)  -0.195  (0.110)  -0.208  (0.085)  0.027  (0.825)  -0.331  (0.004)  -0.209  (0.084)  0.361  (0.002)  -0.222  (0.063)  -0.145  (0.238)  -0.327  (0.005)  -0.132  (0.281)  -0.094  (0.446)  0.012  (0.918) | -0.005  (0.962)  -0.097  (0.462)  0.140  (0.294)  0.195  (0.127)  0.227  (0.075)  -0.019  (0.887)  0.030  (0.822)  0.232  (0.070)  0.163  (0.215)  0.133  (0.323)  0.142  (0.289)  0.099  (0.458)  0.275  (0.033)  0.031  (0.821)  -0.369  (0.007)  0.080  (0.535)  0.116  (0.394)  0.272  (0.034)  0.162  (0.219)  -0.005  (0.962)  -0.063  (0.627) | 0.053  (0.661)  -0.062  (0.611)  -0.016  (0.894)  0.416  (<0.001)  0.175  (0.138)  -0.164  (0.163)  -0.032  (0.794)  0.457  (<0.001)  0.466  (<0.001)  0.258  (0.025)  0.174  (0.138)  0.027  (0.824)  0.433  (<0.001)  0.172  (0.142)  -0.352  (0.002)  0.156  (0.185)  -0.007  (0.950)  0.306  (0.007)  0.183  (0.119)  -0.072  (0.549)  -0.185  (0.118) | 0.084  (0.536)  0.012  (0.927)  -0.179  (0.158)  0.381  (0.005)  0.047  (0.730)  -0.148  (0.254)  -0.007  (0.959)  0.376  (0.006)  0.444  (0.002)  0.205  (0.099)  0.079  (0.567)  -0.052  (0.699)  0.359  (0.007)  0.147  (0.254)  -0.119  (0.368)  0.043  (0.755)  -0.203  (0.103)  0.105  (0.428)  -0.016  (0.911)  -0.147  (0.254)  -0.293  (0.022) |

Supplemental Table 3

Page 6

Correlations of lipid species with different insulin sensitivity indices

| Lipid species | HOMA-IR | GSI | ISI | DI |
| --- | --- | --- | --- | --- |
|  | Spearman’s nonparametric rank correlation coefficient  (Significance) | | | |
| **Phosphatidylcholines (PC)**  PC 40:6  PC 40:7  PC 40:8  **Lysophosphatidylcholines**  **(LPC)**  LPC 14:0  LPC 16:0  LPC 16:1  LPC 17:0  LPC 18:0  LPC 18:1  LPC 18:2  LPC 18:3  LPC 20:3  LPC 20:4  LPC 20:5  LPC 22:5  LPC 22:6  **Lysophosphatidyl-**  **ethanolamines (LPE)**  LPE 16:0  LPE 18:0  LPE 18:1 | -0.105  (0.400)  -0.402  (0.000)  -0.079  (0.520)  0.096  (0.437)  -0.471  (<0.001)  -0.137  (0.268)  -0.333  (0.004)  -0.411  (<0.001)  -0.522  (<0.001)  -0.499  (<0.001)  -0.272  (0.022)  0.246  (0.039)  -0.438  (<0.001)  -0.120  (0.326)  -0.412  (<0.001)  -0.425  (<0.001)  -0.392  (<0.001)  -0.465  (<0.001)  -0.392  (<0.001) | 0.081  (0.533)  0.290  (0.025)  0.092  (0.475)  0.009  (0.946)  0.417  (0.002)  0.063  (0.627)  0.226  (0.074)  0.292  (0.024)  0.480  (<0.001)  0.447  (<0.001)  0.345  (0.010)  0.144  (0.282)  0.358  (0.008)  0.196  (0.125)  0.297  (0.023)  0.407  (0.002)  0.409  (0.002)  0.425  (0.001)  0.466  (<0.001) | 0.119  (0.322)  0.372  (<0.001)  -0.112  (0.349)  -0.085  (0.482)  0.465  (<0.001)  0.078  (0.517)  0.329  (0.003)  0.438  (<0.001)  0.525  (<0.001)  0.602  (<0.001)  0.228  (0.051)  0.282  (0.013)  0.402  (<0.001)  0.190  (0.108)  0.261  (0.023)  0.471  (<0.001)  0.316  (0.005)  0.437  (<0.001)  0.438  (<0.001) | 0.087  (0.531)  0.127  (0.332)  -0.253  (0.047)  0.002  (0.989)  0.284  (0.025)  -0.087  (0.531)  0.241  (0.056)  0.323  (0.012)  0.283  (0.025)  0.435  (0.002)  0.075  (0.591)  0.211  (0.092)  0.220  (0.080)  0.065  (0.639)  -0.030  (0.823)  0.245  (0.052)  0.195  (0.119)  0.180  (0.157)  0.317  (0.014) |

Supplemental Table 3

Page 7

Correlations of lipid species with different insulin sensitivity indices

| Lipid species | HOMA-IR | GSI | ISI | DI |
| --- | --- | --- | --- | --- |
|  | Spearman’s nonparametric rank correlation coefficient  (Significance) | | | |
| **Lysophosphatidyl-**  **ethanolamines (LPE)**  LPE 18:2  LPE 20:4  LPE 22:6  **Ether-linked phospha-**  **tidylcholines (PC O-)**  PC O-32:0  PC O-32:1  PC O-34:1  PC O-34:2  PC O-34:3  PC O-36:2  PC O-36:3  PC O-36:4  PC O-36:5  PC O-36:6  PC O-38:4  PC O-38:5  PC O-38:6  PC O-38:7  PC O-40:7 | -0.387  (<0.001)  -0.394  (<0.001)  -0.369  (0.001)  -0.354  (0.002)  -0.360  (0.002)  -0.451  (<0.001)  -0.379  (<0.001)  -0.519  (<0.001)  -0.451  (<0.001)  -0.381  (<0.001)  -0.301  (0.010)  -0.416  (<0.001)  -0.127  (0.297)  -0.240  (0.044)  -0.445  (<0.001)  -0.372  (0.001)  -0.305  (0.009)  -0.331  (0.004) | 0.368  (0.007)  0.287  (0.026)  0.398  (0.003)  0.259  (0.044)  0.199  (0.120)  0.310  (0.016)  0.305  (0.018)  0.316  (0.015)  0.348  (0.009)  0.270  (0.035)  0.213  (0.094)  0.277  (0.033)  0.127  (0.345)  0.114  (0.402)  0.333  (0.012)  0.273  (0.034)  0.239  (0.063)  0.276  (0.033) | 0.474  (<0.001)  0.264  (0.022)  0.345  (0.002)  0.448  (<0.001)  0.411  (<0.001)  0.511  (<0.001)  0.499  (<0.001)  0.592  (<0.001)  0.564  (<0.001)  0.492  (<0.001)  0.333  (0.003)  0.367  (0.001)  0.200  (0.089)  0.301  (0.008)  0.418  (<0.001)  0.403  (<0.001)  0.311  (0.006)  0.379  (<0.001) | 0.413  (0.003)  0.157  (0.222)  0.175  (0.166)  0.244  (0.053)  0.258  (0.042)  0.334  (0.011)  0.258  (0.042)  0.402  (0.003)  0.359  (0.007)  0.301  (0.019)  0.123  (0.349)  0.170  (0.181)  0.171  (0.180)  0.229  (0.069)  0.180  (0.157)  0.142  (0.270)  0.154  (0.233)  0.211  (0.092) |

Supplemental Table 3

Page 8

Correlations of lipid species with different insulin sensitivity indices

| Lipid species | HOMA-IR | GSI | ISI | DI |
| --- | --- | --- | --- | --- |
|  | Spearman’s nonparametric rank correlation coefficient  (Significance) | | | |
| **Phosphatidylinositoles (PI)**  PI 34:1  PI 34:2  PI 36:1  PI 36:2  PI 36:3  PI 36:4  PI 38:4  PI 38:5  PI 38:6  PI 40:5  PI 40:6  **Phosphatidylethanol-**  **Amines (PE)**  PE 34:1  PE 34:2  PE 36:2  PE 36:3  PE 36:4  PE 38:4  PE 38:5  PE 38:6  PE 40:6 | 0.253  (0.035)  0.063  (0.619)  0.053  (0.680)  -0.195  (0.107)  -0.150  (0.224)  0.039  (0.755)  -0.199  (0.100)  -0.517  (<0.001)  -0.050  (0.691)  0.103  (0.404)  0.034  (0.784)  0.149  (0.227)  0.033  (0.790)  0.091  (0.460)  -0.007  (0.945)  0.061  (0.631)  0.132  (0.281)  0.024  (0.842)  0.098  (0.431)  0.236  (0.046) | -0.227  (0.075)  -0.109  (0.420)  -0.051  (0.695)  0.105  (0.431)  0.037  (0.783)  -0.143  (0.284)  0.093  (0.471)  0.323  (0.014)  0.012  (0.933)  -0.188  (0.141)  -0.042  (0.752)  -0.016  (0.911)  0.023  (0.868)  -0.020  (0.884)  0.109  (0.420)  0.009  (0.946)  -0.084  (0.518)  0.086  (0.508)  0.027  (0.846)  -0.105  (0.431) | -0.218  (0.063)  -0.021  (0.864)  0.101  (0.401)  0.252  (0.028)  0.112  (0.349)  -0.207  (0.079)  0.030  (0.806)  0.437  (<0.001)  -0.062  (0.611)  -0.210  (0.073)  0.044  (0.720)  -0.358  (0.001)  -0.114  (0.341)  -0.083  (0.490)  0.037  (0.764)  -0.265  (0.021)  -0.367  (0.001)  -0.284  (0.013)  -0.289  (0.011)  -0.392  (<0.001) | -0.169  (0.183)  0.013  (0.927)  0.064  (0.639)  0.144  (0.262)  0.099  (0.461)  -0.206  (0.098)  -0.080  (0.562)  0.136  (0.288)  -0.185  (0.144)  -0.279  (0.027)  0.001  (0.989)  -0.300  (0.019)  -0.034  (0.800)  0.012  (0.927)  0.081  (0.556)  -0.239  (0.058)  -0.323  (0.012)  -0.342  (0.010)  -0.308  (0.017)  -0.321  (0.013) |

Supplemental Table 3

Page 9

Correlations of lipid species with different insulin sensitivity indices

| Lipid species | HOMA-IR | GSI | ISI | DI |
| --- | --- | --- | --- | --- |
|  | Spearman’s nonparametric rank correlation coefficient  (Significance) | | | |
| **Phosphatidylethanol-**  **amines (PE)**  PE 40:7  **Ether-linked Phosphatidyl-**  **ethanolamines (PE O-)**  PE O-34:3  PE O-36:3  PE O-36:4  PE O-36:5  PE O-36:6  PE O-38:4  PE O-38:5  PE O-38:6  PE O-38:7  PE O-40:7  PE O-40:8  **Sphingomyelins (SM)**  SM 32:1:1  SM 32:2:1  SM 33:1:1  SM 34:1:1  SM 34:2:1 | -0.123  (0.317)  -0.212  (0.079)  -0.280  (0.019)  -0.203  (0.092)  -0.153  (0.216)  -0.026  (0.825)  -0.167  (0.173)  -0.256  (0.032)  -0.253  (0.035)  -0.205  (0.089)  -0.270  (0.023)  -0.247  (0.039)  -0.247  (0.039)  -0.247  (0.039)  -0.288  (0.015)  -0.442  (<0.001)  -0.484  (<0.001) | 0.193  (0.130)  0.192  (0.132)  0.199  (0.120)  0.164  (0.215)  0.126  (0.348)  0.103  (0.439)  0.087  (0.508)  0.178  (0.172)  0.249  (0.053)  0.247  (0.055)  0.264  (0.041)  0.294  (0.024)  0.164  (0.214)  0.072  (0.580)  0.172  (0.187)  0.293  (0.024)  0.293  (0.024) | -0.062  (0.611)  0.345  (0.002)  0.379  (<0.001)  0.330  (0.003)  0.211  (0.072)  0.142  (0.229)  0.195  (0.099)  0.277  (0.015)  0.301  (0.008)  0.240  (0.038)  0.337  (0.003)  0.340  (0.003)  0.405  (<0.001)  0.349  (0.002)  0.364  (0.001)  0.530  (<0.001)  0.558  (<0.001) | -0.211  (0.092)  0.329  (0.011)  0.315  (0.014)  0.251  (0.047)  0.210  (0.093)  0.145  (0.259)  0.237  (0.059)  0.227  (0.071)  0.180  (0.157)  0.157  (0.222)  0.272  (0.032)  0.251  (0.047)  0.363  (0.007)  0.339  (0.011)  0.331  (0.012)  0.401  (0.003)  0.396  (0.003) |

Supplemental Table 3

Page 10

Correlations of lipid species with different insulin sensitivity indices

| Lipid species | HOMA-IR | GSI | ISI | DI |
| --- | --- | --- | --- | --- |
|  | Spearman’s nonparametric rank correlation coefficient  (Significance) | | | |
| **Sphingomyelins (SM)**  SM 35:1:1  SM 36:1:1  SM 36:2:1  SM 36:3:1  SM 37:1:1  SM 38:1:1  SM 38:2:1  SM 39:1:1  SM 39:2:1  SM 40:1:1  SM 40:2:1  SM 40:3:1  SM 41:1:1  SM 41:2:1  SM 41:3:1  SM 42:1:1  SM 42:2:1  SM 42:3:1  SM 42:4:1  SM 43:2:1 | -0.237  (0.046)  -0.301  (0.010)  -0.489  (<0.001)  -0.243  (0.041)  -0.112  (0.362)  -0.333  (0.004)  -0.489  (<0.001)  -0.165  (0.176)  -0.207  (0.086)  -0.257  (0.032)  -0.465  (<0.001)  -0.339  (0.003)  -0.351  (0.002)  -0.383  (<0.001)  -0.308  (0.008)  -0.132  (0.281)  -0.475  (<0.001)  -0.511  (<0.001)  -0.523  (<0.001)  -0.227  (0.058) | 0.077  (0.550)  0.155  (0.243)  0.238  (0.065)  0.098  (0.459)  -0.011  (0.937)  0.261  (0.043)  0.338  (0.011)  0.081  (0.533)  0.107  (0.429)  0.154  (0.243)  0.329  (0.012)  0.095  (0.464)  0.218  (0.085)  0.274  (0.034)  0.130  (0.334)  0.039  (0.773)  0.329  (0.012)  0.348  (0.009)  0.237  (0.065)  0.137  (0.306) | 0.320  (0.005)  0.372  (0.001)  0.478  (<0.001)  0.296  (0.009)  0.171  (0.145)  0.422  (<0.001)  0.451  (<0.001)  0.259  (0.024)  0.258  (0.025)  0.343  (0.002)  0.525  (<0.001)  0.389  (<0.001)  0.396  (<0.001)  0.425  (<0.001)  0.375  (<0.001)  0.184  (0.119)  0.489  (<0.001)  0.536  (<0.001)  0.484  (<0.001)  0.309  (0.007) | 0.341  (0.011)  0.302  (0.019)  0.364  (0.007)  0.230  (0.069)  0.219  (0.080)  0.308  (0.017)  0.320  (0.013)  0.267  (0.036)  0.303  (0.019)  0.295  (0.021)  0.360  (0.007)  0.350  (0.009)  0.271  (0.033)  0.329  (0.012)  0.358  (0.007)  0.098  (0.468)  0.276  (0.029)  0.297  (0.020)  0.305  (0.018)  0.220  (0.080) |
