# Supplementary material for: Detection of Independent Associations of Plasma Lipidomic Parameters with Insulin Sensitivity Indices Using Data Mining Methodology
Source: PLoS One. 2016 Oct 13;11(10):e0164173. doi: 10.1371/journal.pone.0164173 (PMC5063331; doi:10.1371/journal.pone.0164173)
Supplement: S4 Table — (DOCX) [file pone.0164173.s006.docx]

Supplemental Table 4

Insulin sensitivity/ resistance indices

| Index | Formula |
| --- | --- |
| HOMA-IR  GSI  ISI  DI | PG0 x Ins0 /154.8  Glucose: mmol/l  Insulin: pmol/l  Kazama et al. Diabetes Res Clin Pract 79(2008)24-30  🠦 autoregressive model  🠦 GSI calculator: http://www18.ocn.ne.jp/~ogsi/  (75000 + [(PG0 – PG120) x 0.19 x body weight]) / 120  (PG0-PG120) / 2 x log [(Ins0+Ins120) /2]  Glucose: mg/dl  Insulin: µU/ml  (1283 + 1.829 x Ins30 -138.7 x PG30 + 3.772 x Ins0) x ISI  Glucose: mmol/l  Insulin: pmol/l |

For comparability with results published elsewhere, different units for glucose and insulin were applied.
